# Impact of ECOG performance status 2 participants on outcomes of pivotal cancer clinical trials: a meta-analysis and meta-regression

**DOI:** 10.1016/j.esmoop.2026.106065

**Published:** 2026-02-02

**Authors:** G.M. Iannantuono, T. Giovagnoli, L. Mastrantoni, B. Gyawali, C.S. Floudas, S. Sganga, D. Giannarelli, M. Filetti, A. Spinazzola, F. Lo Bianco, E. Giudice, A. Vitale, J.L. Gulley, P. Navarra, E. Bria, G. Daniele

**Affiliations:** 1 Phase 1 Unit, Fondazione Policlinico Universitario Agostino Gemelli IRCCS, Rome, Italy; 2 Comprehensive Cancer Center, Medical Oncology Department, Fondazione Policlinico Universitario Agostino Gemelli IRCCS, Rome, Italy; 3 Faculty of Medicine and Surgery, Università Cattolica del Sacro Cuore, Rome, Italy; 4 Division of Cancer Care and Epidemiology, Queen’s Cancer Research Institute, Kingston, Canada; 5 Department of Oncology, Queen’s University, Kingston, Canada; 6 Public Health Sciences, Queen’s University, Kingston, Canada; 7 Center for Immuno-Oncology, Center for Cancer Research, National Cancer Institute, National Institutes of Health, Bethesda, USA; 8 Facility of Epidemiology and Biostatistics, Fondazione Policlinico Universitario Agostino Gemelli IRCCS, Rome, Italy; 9 Division of Gynecologic Oncology, Humanitas San Pio X, Milan, Italy; 10 Section of Pharmacology, Department of Healthcare Surveillance and Bioethics, Catholic University Medical School, Fondazione Policlinico Universitario Agostino Gemelli IRCCS, Rome, Italy; 11 Medical Oncology Unit, Ospedale Isola Tiberina-Gemelli Isola, Rome, Italy

**Keywords:** performance status, pivotal clinical trial, eligibility criteria, solid tumor, meta-analysis

## Abstract

**Background:**

Although patients with Eastern Cooperative Oncology Group performance status (PS) of 2 constitute a significant proportion of the cancer population, they are often excluded from pivotal clinical trials owing to presumed higher risks of treatment effect dilution, toxicity, and lower compliance. Here, we conducted a systematic review and meta-analysis to evaluate the impact of including PS 2 participants on efficacy and safety outcomes in pivotal cancer clinical trials.

**Materials and methods:**

We searched the ‘Oncology/Hematologic Malignancies Approval Notifications’ and ‘Drugs@FDA' databases for clinical trials supporting ‘Food and Drug Administration' anticancer drug approvals from 1 January 2009 to 31 December 2024. Eligible studies were randomized phase III clinical trials of systemic therapies for metastatic solid tumors permitting the inclusion of PS 2 participants. We assessed efficacy outcomes [progression-free survival (PFS) and overall survival (OS)] and safety outcomes [occurrence of any-grade adverse events (AEs), high-grade AEs, serious AE (SAEs), AE-related deaths, and treatment modifications] in the included studies.

**Results:**

Thirty-six trials were included. In subgroup analyses, no statistically significant differences were found between PS 2 and PS ≤1 participants for PFS [hazard ratio (HR) 0.45, 95% confidence interval (CI) 0.30-0.69 versus HR 0.52, 95% CI 0.41-0.66, *P* = 0.59] and OS (HR 0.81, 95% CI 0.68-0.97 versus HR 0.71, 95% CI 0.66-0.77, *P* = 0.18). In meta-regression analyses, no significant associations were found for efficacy outcomes. However, a higher proportion of PS 2 participants was significantly associated with an increased risk of SAEs, AE-related deaths, and treatment discontinuations.

**Conclusions:**

Although PS 2 participants showed a greater propensity to serious toxicity, no significant differences in efficacy outcomes were observed compared with those with PS ≤1. Our results support the inclusion of PS 2 participants in clinical trials, as their exclusion limits the generalizability of results.

## Highlights

• No significant differences for efficacy outcomes were observed between ECOG PS 2 and PS ≤1 participants in pivotal trials.
• Higher ECOG PS 2 representation was associated with increased SAEs, treatment discontinuations, and AE-related deaths.
• Inclusion of ECOG PS 2 participants in clinical trials is warranted to enhance the generalizability of study outcomes.

## Introduction

Performance status (PS) measures patients’ functional status based on their ability to manage self-care, conduct activities of daily living, and execute physical tasks.^1^ The attribution of a PS score relies on the use of scales introduced initially in 1948 to assess the effects of chemotherapy in cancer patients. By the 1970s, however, the application of PS evolved from evaluating the efficacy of anticancer interventions to stratifying participants’ functional capabilities in clinical trials, with the objective of minimizing the heterogeneity of the enrolled population.^2^ Multiple PS scales are currently validated to categorize patients according to their functional status, such as the Eastern Cooperative Oncology Group (ECOG) PS scale and the Karnofsky Performance Scale (KPS). Lower KPS values and higher ECOG PS scores are indicative of poor functional status.^3^^,^^4^

Although patients with poor functional status constitute a substantial proportion of the general cancer population, it has been described that they are often excluded from pivotal clinical trials, mainly due to a presumed higher probability of treatment effect dilution and side-effects as well as lower compliance.^5^ In this context, several studies have investigated whether the efficacy of systemic anticancer therapies varies across patients when stratified according to their PS.^6^, ^7^, ^8^ In 2017, Cheng et al. reported the results of a meta-analysis including cancer patients enrolled in pivotal clinical trials and treated with novel chemotherapeutic agents and targeted therapies, finding no differences in terms of survival between participants with ‘excellent’ and ‘reduced’ PS.^6^ Notably, the distinction between patients with ‘excellent’ and ‘reduced’ PS did not follow a predefined rule but was based on how subgroup outcomes were reported in the included studies.^6^ In 2018 and 2020, two additional meta-analyses assessed the survival outcomes of cancer patients treated with immune checkpoint inhibitors, and similarly found no difference in survival benefit between participants with ECOG PS 0 and those with ECOG PS 1-2.^7^^,^^8^

Despite providing important insights into the association between PS and the efficacy of anticancer treatments, previous meta-analyses have often grouped patients with ECOG PS 1 and PS 2, comparing them with those classified as ECOG PS 0.^6^, ^7^, ^8^ This methodological approach likely reflects the limited enrollment of patients with ECOG PS 2 in clinical trials, who are therefore frequently combined with those with ECOG PS 1 in subgroup analyses.^5^ However, the level of functional impairment associated with ECOG PS 2 is significantly different from that observed in patients with PS 1, and this aggregation may mask potentially relevant differences in therapeutic outcomes. In this context, we aimed to assess the impact of including ECOG PS 2 participants on the efficacy and safety outcomes of pivotal phase III cancer clinical trials, compared with those with ECOG PS ≤1.

## Materials and methods

We conducted a systematic review and meta-analysis in accordance with the Preferred Reporting Items for Systematic reviews and Meta-Analyses (PRISMA) statement (Supplementary Table S1, available at https://doi.org/10.1016/j.esmoop.2026.106065).^9^ We designed and registered a protocol for this systematic review in the International Prospective Register of Systematic Reviews PROSPERO (CRD420251062013).This study did not involve human participants, and therefore no institutional review board approval or informed consent was required.

### Search strategy and eligibility criteria

We conducted a comprehensive search in the ‘Oncology (Cancer)/Hematologic Malignancies Approval Notifications’ section of the United States Food and Drug Administration (FDA) website^10^ and in the ‘Drugs@FDA' database^11^ for clinical trials leading to drug approvals from 1 January 2009 to 31 December 2024. We included phase III clinical trials that: (i) led to drug approvals by the FDA; (ii) enrolled participants affected by metastatic solid tumors and treated with anticancer therapies; and (iii) permitted the inclusion of participants with ECOG PS ≥2. We excluded the following types of studies: (i) phase I and II clinical trials; (ii) single-arm phase III clinical trials; (iii) phase III clinical trials excluding participants with ECOG PS ≥2; and (iv) phase III clinical trials enrolling participants affected by hematologic malignancies.

### Study selection and data collection

The study selection was independently carried out by two authors (GMI and TG) using a two-stage process. Firstly, the content available in each FDA approval notification was reviewed, and the referenced clinical trials were selected. Subsequently, clinical trials’ protocols and manuscripts were evaluated in accordance with the eligibility criteria. An agreement between the two authors was required for inclusion and exclusion at both stages. Disagreements were discussed with an additional author (GD) and resolved by consensus.

### Definition of outcomes

From the included clinical trials, two authors (GMI and TG) independently extracted the study characteristics (first author, year of publication, enrolled population, class of drugs, details of treatment arms, primary endpoints), baseline participant characteristics (primary tumor site and PS), and study outcomes related to both efficacy and safety. For efficacy outcomes, we extracted hazard ratios (HRs) with 95% confidence intervals (CIs) for progression-free survival (PFS) and overall survival (OS) in the overall population and separately for participants with ECOG PS ≤1 and PS 2. Regarding safety outcomes, we assessed the proportions of participants experiencing any-grade adverse events (AEs), high-grade (grade ≥3) AEs, serious AEs (SAEs), and AE-related deaths (Supplementary Table S2, available at https://doi.org/10.1016/j.esmoop.2026.106065). We also evaluated the proportion of participants who experienced treatment modifications (dose reductions, dose discontinuations, or dose interruptions). In clinical trials using different PS scales (e.g. KPS, the World Health Organization scale, or the Gynecologic Oncology Group scale), we converted PS scores to the ECOG PS scale based on available literature^6^ (Supplementary Table S3, available at https://doi.org/10.1016/j.esmoop.2026.106065). As in the study selection process, in cases of inconsistencies, an additional author (GD) was involved in the discussion, and disagreements were resolved by consensus.

### Risk-of-bias assessment

Two authors (GMI and TG) assessed the risk of bias (RoB) of the included studies using the revised Cochrane RoB tool for randomized trials (RoB 2).^12^ An overall RoB was attributed to each included clinical trial. Inconsistencies were resolved by consensus after a discussion with an additional author (GD).

### Statistical analyses

We summarized the study and participant characteristics using descriptive statistics and reported the results as absolute numbers and proportions, along with interquartile ranges (IQRs) and standard deviations, as appropriate. Pooled estimates of HR with their 95% CI for OS and PFS were calculated using the random-effects model using the inverse-variance method. Between-study variance (τ^2^) was estimated using the restricted maximum likelihood estimator. CIs for τ^2^ and τ were obtained using the Q-profile method. In contrast, pooled estimates of proportions for safety outcomes were obtained using a generalized linear mixed model (a random-intercept logistic regression model). Heterogeneity between studies was assessed using the inconsistency index (*I*^2^).^13^ Although publication bias was inherently expected given the inclusion of only clinical trials that led to drug approvals, we evaluated its presence using funnel plots and, when applicable, Egger’s test.^14^^,^^15^

As a central element of our analytic framework, we conducted a subgroup analysis to investigate whether there was a significant difference in survival outcomes between participants with PS ≤1 and those with PS 2. When clinical trials reported HR for PS 0 and PS 1 separately, a pooled estimate of the two HRs was used for the subgroup analysis. Exploratory subgroup analyses were also carried out to evaluate differences between PS ≤1 and PS 2 participants within strata defined by tumor type and drug class. In addition, to further investigate the impact of baseline PS on efficacy and safety outcomes, we carried out meta-regression analyses using the study-level proportions of patients with PS 0, PS 1, and PS 2 in the included clinical trials as independent variables. Firstly, we carried out univariable meta-regression models with each PS proportion (PS 0, PS 1, and PS 2) as individual predictors. We then constructed a multivariable model that included the proportions of patients with PS 1 and PS 2 to evaluate their independent contributions to treatment outcomes. As a sensitivity analysis, we also carried out meta-regression analyses using arcsine square root-transformed proportions to stabilize variance and account for the bounded nature of proportions, particularly given the low expected prevalence of PS 2 participants.^16^^,^^17^ We also evaluated the effect of sample size on efficacy outcomes by including the total number of study participants in the multivariable meta-regression model. Collinearity among covariates included in the models was assessed using variance inflation factors. Statistical analyses were carried out using the ‘RStudio: integrated development environment for R' (version 4.2.2 - Posit software, PBC - Boston, MA), with primary analyses conducted using the ‘meta' and ‘metafor' packages; a complete list of additional packages is provided in Supplementary Material, available at https://doi.org/10.1016/j.esmoop.2026.106065.

## Results

### Study selection and baseline characteristics

The results of the literature search and the study selection process are reported in the PRISMA flow diagram (Supplementary Figure S1, available at https://doi.org/10.1016/j.esmoop.2026.106065). A total of 36 clinical trials were deemed eligible for inclusion^18^, ^19^, ^20^, ^21^, ^22^, ^23^, ^24^, ^25^, ^26^, ^27^, ^28^, ^29^, ^30^, ^31^, ^32^, ^33^, ^34^, ^35^, ^36^, ^37^, ^38^, ^39^, ^40^, ^41^, ^42^, ^43^, ^44^, ^45^, ^46^, ^47^, ^48^, ^49^, ^50^, ^51^, ^52^, ^53^ (Supplementary Tables S4 and S5, available at https://doi.org/10.1016/j.esmoop.2026.106065). Since two clinical trials (NCT03914612 and NCT01847274) reported outcomes separately for two predefined patient cohorts, we considered each cohort as an independent study.^20^^,^^34^ Eight clinical trials did not report a breakdown of participants according to PS. In the remaining studies, the median proportions (IQR) of patients with PS 0, PS 1, and PS 2 were 49.4% (41.5%-64.1%), 44.3% (32.0%-53.2%), and 4.1% (2.7%-6.4%), respectively. The RoB was low across all included trials (Supplementary Figures S2 and S3, available at https://doi.org/10.1016/j.esmoop.2026.106065). Results of the publication bias assessment are reported in Supplementary Figures S4-S12, available at https://doi.org/10.1016/j.esmoop.2026.106065.

### Efficacy outcomes

**Figure 1:**
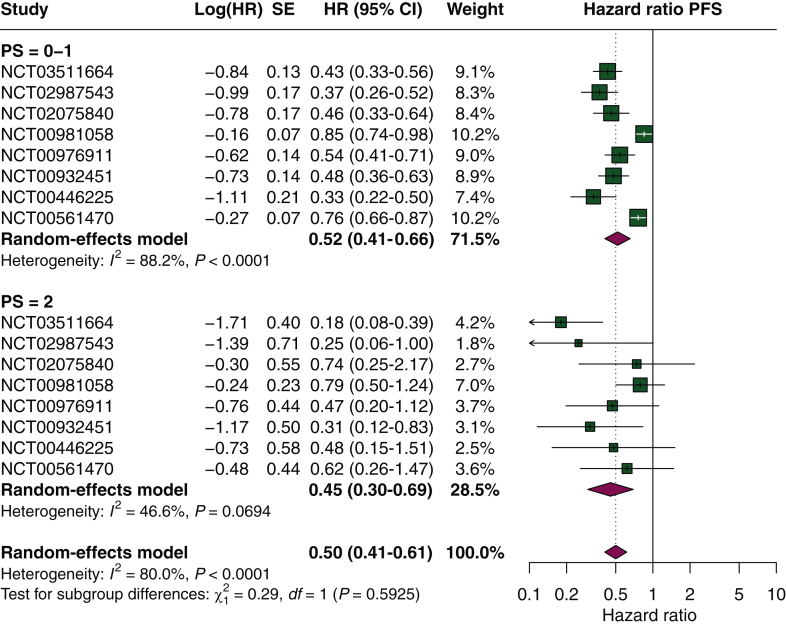
**Subgroup analysis of PFS according to performance status.** CI, confidence interval; HR, hazard ratio; PFS, progression-free survival; SE, standard error.

**Figure 2:**
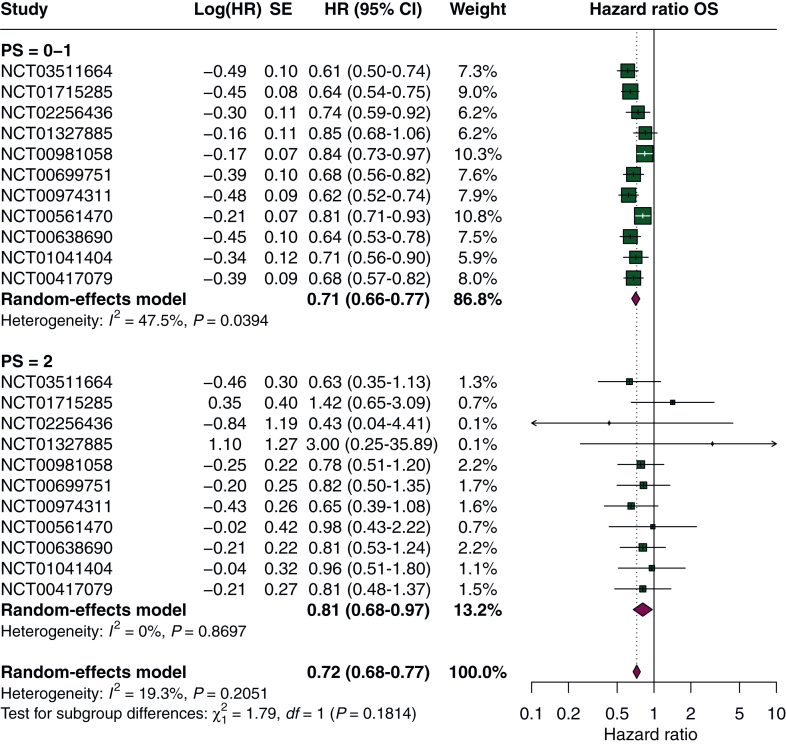
**Subgroup analysis of OS according to performance status.** CI, confidence interval; HR, hazard ratio; OS, overall survival; SE, standard error.

A total of 16 clinical trials reported survival outcomes for PS 2 participants: 3 reported both PFS and OS, 5 reported PFS only, and 8 reported OS only (Supplementary Table S4, available at https://doi.org/10.1016/j.esmoop.2026.106065). Among trials reporting separated outcomes for PS 0 and PS 1 participants, pooled estimates for PS ≤1 were derived from six studies for PFS and three studies for OS (Supplementary Table S6, available at https://doi.org/10.1016/j.esmoop.2026.106065). For PFS, the pooled HR was 0.52 (95% CI 0.41-0.66, *I*^2^ = 88%) for PS ≤1 participants and 0.45 (95% CI 0.30-0.69, *I*^2^ = 45%) for those with PS 2, with no statistically significant difference between subgroups (*P* = 0.59) (Figure 1). For OS, pooled HRs were 0.71 (95% CI 0.66-0.77, *I*^2^ = 48%) for participants with PS ≤1 and 0.81 (95% CI 0.68-0.97, *I*^2^ = 0%) for those with PS 2 (*P* = 0.18) (Figure 2). In exploratory subgroup analyses stratified by tumor type and drug class, no conclusive evidence of effect modification by PS was observed for efficacy outcomes (Supplementary Figures S13-S16, available at https://doi.org/10.1016/j.esmoop.2026.106065). In meta-regression analyses, no statistically significant associations with efficacy outcomes were observed for PS 2 and PS 0 participants. For PS 1 participants, a higher proportion was significantly associated with reduced PFS benefit in both univariable and multivariable models. No significant associations with OS were observed in models using raw proportions; however, a significant association with reduced OS benefit was observed in the univariable arcsine-transformed model (Table 1, Supplementary Figures S17 and S18 and Tables S7-S10, available at https://doi.org/10.1016/j.esmoop.2026.106065). No collinearity issues were detected (Supplementary Table S18, available at https://doi.org/10.1016/j.esmoop.2026.106065).

**Table 1:** Meta-regression models for efficacy outcomes using proportions of participants according to performance status

Outcome	Model	Variable^a^	Estimate (β)	*P* value	95% CI	
PFS	Univariable (*n* = 27)	Intercept	−0.3556	0.3034	−1.0329	0.3216
		PS 0 participants	−0.0066	0.3082	−0.0194	0.0061
	Univariable (*n* = 27)	Intercept	−1.3501	<0.0001	−1.8519	−0.8482
		**PS 1 participants**	**0.0156**	**0.0069**	**0.0043**	**0.0269**
	Univariable (*n* = 33)	Intercept	−0.8498	<0.0001	−1.1113	−0.5883
		PS 2 participants	0.0317	0.1796	−0.0146	0.0781
	Multivariable (*n* = 27)	Intercept	−1.4005	<0.0001	−1.9184	−0.8826
		**PS 1 participants**	**0.0193**	**0.0086**	**0.0049**	**0.0337**
		PS 2 participants	−0.0251	0.4159	−0.0856	0.0354
OS	Univariable (*n* = 23)	Intercept	−0.1949	0.3422	−0.5972	0.2073
		PS 0 participants	−0.0017	0.6998	−0.0101	0.0068
	Univariable (*n* = 23)	Intercept	−0.6083	0.002	−0.9937	−0.2229
		PS 1 participants	0.0072	0.0763	−0.0008	0.0151
	Univariable (*n* = 31)	Intercept	−0.3762	<0.0001	−0.5188	−0.2336
		PS 2 participants	0.0134	0.2497	−0.0094	0.0361
	Multivariable (*n* = 23)	Intercept	−0.5863	0.0052	−0.9973	−0.1753
		PS 1 participants	0.0058	0.2658	−0.0044	0.0161
		PS 2 participants	0.0085	0.6352	−0.0267	0.0437

Covariates with statistically significant values are shown in bold (*P* < 0.05).

CI, confidence interval; OS, overall survival; PFS, progression-free survival; PS, performance status.

a Proportion of participants enrolled in clinical trials according to performance status.

### Safety outcomes

For PS 2 participants, the meta-regression analyses showed a statistically significant association between a higher proportion of patients and an increased occurrence of SAEs and treatment discontinuations at univariable analyses (with both raw and arcsine-transformed proportions) and AE-related deaths in the only multivariable arcsine-transformed model (Tables 2 and 3, Supplementary Figures S21, S24, and S25 and Tables S13, S16, and S17, available at https://doi.org/10.1016/j.esmoop.2026.106065). No significant associations were found for any-grade AEs, high-grade AEs, dose interruptions, and dose reductions (Tables 2 and 3, Supplementary Figures S19, S20, S22, and S23 and Tables S11, S12, S14, and S15, available at https://doi.org/10.1016/j.esmoop.2026.106065). For PS 1 participants, a higher proportion was significantly associated with an increased risk of SAEs in both univariable and multivariable models (Table 2, Supplementary Figure S21 and Table S13, available at https://doi.org/10.1016/j.esmoop.2026.106065). Additionally, significant positive β coefficients were also found for dose discontinuations in univariable models and dose reductions in the only multivariable arcsine-transformed model (Table 3, Supplementary Figures S23-S25 and Tables S15 and S16, available at https://doi.org/10.1016/j.esmoop.2026.106065). For PS 0 participants, a higher proportion was significantly associated with a reduced occurrence of AE-related deaths (also confirmed in the arcsine-transformed model) and an increased frequency of dose reductions across all models (Tables 2 and 3, Supplementary Figures S23 and S25 and Tables S15 and S17, available at https://doi.org/10.1016/j.esmoop.2026.106065). No collinearity issues were detected (Supplementary Table S18, available at https://doi.org/10.1016/j.esmoop.2026.106065).

**Table 2:** Meta-regression models for safety outcomes using proportions of participants according to performance status

Outcome	Model	Variable^a^	Estimate (β)	*P* value	95% CI	
Any-grade AEs	Univariable (*n* = 23)	Intercept	4.062	0.0001	2.2703	5.8536
		PS 0 participants	−0.0126	0.4657	−0.048	0.0227
	Univariable (*n* = 23)	Intercept	2.7391	0.0103	0.7186	4.7596
		PS 1 participants	0.0153	0.4632	−0.0273	0.0579
	Univariable (*n* = 29)	Intercept	3.0137	<0.0001	2.3039	3.7235
		PS 2 participants	0.0746	0.2023	−0.0425	0.1916
	Multivariable (*n* = 23)	Intercept	3.0165	0.0104	0.7902	5.2428
		PS 1 participants	0.0043	0.8758	−0.0526	0.0613
		PS 2 participants	0.0501	0.5561	−0.1244	0.2245
High-grade AEs	Univariable (*n* = 20)	Intercept	0.7612	0.0525	−0.0092	1.5316
		PS 0 participants	−0.0101	0.1868	−0.0255	0.0054
	Univariable (*n* = 20)	Intercept	−0.3588	0.4103	−1.2529	0.5354
		PS 1 participants	0.0135	0.141	−0.0049	0.032
	Univariable (*n* = 26)	Intercept	0.1097	0.455	−0.1884	0.4077
		PS 2 participants	0.0281	0.2393	−0.02	0.0762
	Multivariable (*n* = 20)	Intercept	−0.313	0.519	−1.3159	0.6898
		PS 1 participants	0.0118	0.3335	−0.0132	0.0369
		PS 2 participants	0.0076	0.8327	−0.0675	0.0828
SAEs	Univariable (*n* = 15)	Intercept	−0.1446	0.7163	−0.9852	0.6961
		PS 0 participants	−0.0154	0.0701	−0.0322	0.0015
	Univariable (*n* = 15)	Intercept	−1.9895	<0.0001	−2.5688	−1.4103
		**PS 1 participants**	**0.0258**	**0.0006**	**0.0134**	**0.0381**
	Univariable (*n* = 20)	Intercept	−1.1602	<0.0001	−1.463	−0.8554
		**PS 2 participants**	**0.0609**	**0.0093**	**0.017**	**0.1049**
	Multivariable (*n* = 15)	Intercept	−1.9964	<0.0001	−2.6462	−1.3466
		**PS 1 participants**	**0.0261**	**0.011**	**0.0072**	**0.045**
		PS 2 participants	−0.0016	0.9588	−0.0674	0.0642
AE-related deaths	Univariable (*n* = 22)	Intercept	−1.0412	0.2229	−2.7677	0.6852
		**PS 0 participants**	**−0.0523**	**0.0053**	**−0.0872**	**−0.0175**
	Univariable (*n* = 22)	Intercept	−4.0869	<0.0001	−5.7722	−2.4467
		PS 1 participants	0.0106	0.5395	−0.0248	0.046
	Univariable (*n* = 30)	Intercept	−4.0329	<0.0001	−4.7918	−3.274
		PS 2 participants	0.101	0.099	−0.0202	0.2222
	Multivariable (*n* = 22)	Intercept	−3.8597	<0.0001	−5.4562	−2.2542
		PS 1 participants	−0.0069	0.7427	−0.05	0.0363
		PS 2 participants	0.1235	0.2021	−0.0721	0.319

Covariates with statistically significant values are shown in bold (*P* < 0.05).

AE, adverse event; CI, confidence interval; PS, performance status; SAE, severe adverse event.

a Proportion of participants enrolled in clinical trials according to performance status.

**Table 3:** Meta-regression models for treatment modifications using proportions of participants according to performance status

Outcome	Model	Variable^a^	Estimate (β)	*P* value	95% CI	
Dose reductions	Univariable (*n* = 11)	Intercept	−2.7257	0.0003	−4.2797	−1.1717
		**PS 0 participants**	**0.0352**	**0.0289**	**0.0045**	**0.0658**
	Univariable (*n* = 11)	Intercept	−1.6056	0.0299	−3.0153	−0.196
		PS 1 participants	0.0139	0.338	−0.0172	0.045
	Univariable (*n* = 14)	Intercept	−0.8141	0.0589	−1.6642	0.036
		PS 2 participants	−0.0591	0.4211	−0.2137	0.0955
	Multivariable (*n* = 11)	Intercept	−1.7146	0.0208	−3.0921	−0.3371
		PS 1 participants	0.0246	0.1586	−0.0119	0.0611
		PS 2 participants	−0.0838	0.2524	−0.2406	0.0729
Dose interruptions	Univariable (*n* = 9)	Intercept	−0.2055	0.9026	−4.0372	3.6261
		PS 0 participants	−0.0107	0.7314	−0.0814	0.06
	Univariable (*n* = 9)	Intercept	−2.0356	0.0404	−3.9539	−0.1174
		PS 1 participants	0.0335	0.1267	−0.0122	0.0792
	Univariable (*n* = 11)	Intercept	−0.8585	0.1539	−2.1059	0.3889
		PS 2 participants	0.043	0.7693	−0.2787	0.3647
	Multivariable (*n* = 9)	Intercept	−2.4994	0.0248	−4.5544	−0.4444
		PS 1 participants	0.067	0.0853	−0.0127	0.1466
		PS 2 participants	−0.3344	0.2633	−0.9975	0.3286
Dose discontinuations	Univariable (*n* = 19)	Intercept	−1.4664	0.0033	−2.3718	−0.561
		PS 0 participants	−0.0106	0.2325	−0.0287	0.0075
	Univariable (*n* = 19)	Intercept	−2.7933	<0.0001	−3.5989	−1.9878
		**PS 1 participants**	**0.0182**	**0.0378**	**0.0012**	**0.0353**
	Univariable (*n* = 25)	Intercept	−2.4228	<0.0001	−2.797	−2.0486
		**PS 2 participants**	**0.072**	**0.0194**	**0.0127**	**0.1312**
	Multivariable (*n* = 19)	Intercept	−2.6523	<0.0001	−3.4677	−1.837
		PS 1 participants	0.0107	0.295	−0.0103	0.0317
		PS 2 participants	0.043	0.2363	−0.0311	0.1171

Covariates with statistically significant values are shown in bold (*P* < 0.05).

CI, confidence interval; PS, performance status.

a Proportion of participants enrolled in clinical trials according to performance status.

## Discussion

We conducted a systematic review and meta-analysis to evaluate the impact of enrolling PS 2 participants in phase III clinical trials leading to FDA anticancer drug approvals. For the first time, we analyzed efficacy and safety outcomes reported for PS 2 participants separately and compared them to those achieved by PS ≤1 participants. Our analytic strategy encompasses subgroup and meta-regression analyses. Particularly, subgroup analyses did not reveal any statistically significant differences in survival outcomes between PS 2 and PS ≤1 participants. While PS 2 participants derived a meaningful benefit in terms of PFS, the magnitude of benefit on OS appeared reduced. Several factors may explain these findings. On one hand, this may reflect the greater clinical vulnerability and reduced functional reserve of this subgroup, whose mortality may be influenced by factors unrelated to disease progression.^54^^,^^55^ On the other hand, the reliance on HR may be misleading. Small absolute survival gains in populations with limited baseline survival can translate into a relatively large reduction in HR, potentially overestimating treatment effects in this population.^56^

We then used meta-regression in order to examine whether different PS subgroups were associated with different magnitudes of benefit. Although meta-regression did not find any statistically significant association in the PS 2 subgroup, the positive β coefficients across the models suggested a potentially lower magnitude of benefit as the proportion of these patients with PS 2 increased. The absence of statistical significance is likely attributable to the small sample size in this subgroup and its relatively homogeneous distribution across the studies. Regarding the safety outcomes, the association between a higher proportion of PS 2 participants and increased risk of SAEs, treatment discontinuations, and AE-related deaths confirmed the greater clinical vulnerability of this subgroup. However, these associations (except for AE-related deaths) were not significant in multivariable analyses, suggesting a potential overlap with the effect of PS 1 participants due to the correlation between these groups and the small sample size of PS 2 patients. Moreover, PS 2 participants in clinical trials are often highly selected, with functional impairments related to controlled comorbidities or reversible factors, thereby showing a therapeutic response closer to that of PS 1 patients.

For the PS 1 population, a higher proportion of participants was associated with reduced PFS and OS, as well as higher rates of SAEs and dose reductions. Although these results may suggest clinical heterogeneity in this population, they should be interpreted with caution. For the PS 0 population, the negative β coefficients demonstrated across the meta-regression models suggested a greater benefit with increasing representation of these participants, consistent with lower clinical vulnerability and better treatment tolerance. In this direction, the statistically significant positive β coefficient for dose reduction supports the better baseline functional status of these patients, resulting in a longer treatment duration and, thus, an increased probability of dose modifications over time. Our results suggest that as the PS worsens, efficacy outcomes may decline, while the probability of AEs and reduced adherence increases.

Lastly, the use of PS scales has inherent limitations that warrant discussion. Firstly, the attribution of a PS score is highly subjective, with significant variability among clinicians and also discrepancies between physician and patient self-assessments.^57^ Furthermore, the mischaracterization of PS may have substantial consequences for clinical trial eligibility, since some patients may be considered to have an eligible PS despite having an initial and subtle functional decline. In contrast, other patients may not be judged as good candidates based on their PS despite retaining a reserved capacity to tolerate the experimental treatment.^58^ Consequently, the ‘a priori’ exclusion of PS 2 participants appears paradoxical, as the investigator could easily ‘reclassify’ borderline PS 2 patients as PS 1 and allow them to participate in a clinical trial. Furthermore, PS 2 patients enrolled in clinical trials are often highly selected and therefore may differ from ‘real-world’ PS 2 patients, reinforcing the critical need to integrate experimental data with real-world evidence to fully understand the risks and benefits of anticancer treatments, especially in the most vulnerable populations. Considering the subjectivity and limited precision of PS assessment, clinical trials must include participants across all PS subgroups to obtain data that are truly representative of the population we treat in routine clinical practice.^2^^,^^5^ Although novel, more objective tools have shown promising results in improving the utility of PS assessment, none are ready for implementation in clinical practice.^2^ Therefore, clinical trials should continue to include patients from all PS subgroups to allow the enrollment of a population that is truly representative of routine clinical practice.

### Limitations

We acknowledge that our study has several limitations. Firstly, there is a risk of ecological fallacy when modeling an individual-level variable, such as PS, using study-level aggregated data.^59^ Although PS cannot be considered the sole prognostic factor due to the potential influence of other clinical characteristics, the consistency between subgroup analyses and meta-regression models supports the robustness of our findings.^60^ Secondly, we included only studies supporting regulatory drug approvals, which introduces a risk of publication bias, as deviations from expected outcomes are more likely in positive trials. Since smaller studies may systematically overestimate PFS benefits,^61^ we carried out additional meta-regression analyses including study sample size as a covariate, which confirmed the stability of our PS results.^62^^,^^63^ Thirdly, the small proportion of pivotal clinical trials reporting outcomes for PS 2 participants and the heterogeneity in terms of tumor types, treatments, and lines of therapy limit the generalizability of our results. Lastly, PS 2 participants enrolled in randomized clinical trials leading to FDA approval may not be representative of the broader PS 2 patient population encountered in routine clinical practice, increasing the likelihood of selection bias toward fitter PS 2 patients and potentially leading to an overestimation of efficacy and an underestimation of toxicity in this subgroup.

### Conclusions

Although participants with PS 2 exhibited a higher propensity for serious toxicity, no significant differences in efficacy outcomes were observed compared with those with PS ≤1 in clinical trials that led to FDA approval of anticancer drugs. However, it is important to acknowledge that PS 2 patients enrolled in these trials may not reflect the broader population encountered in routine clinical practice. Overall, our findings support the inclusion of PS 2 patients in clinical trials, as their exclusion restricts the generalizability of results. Furthermore, considering the subjective nature and potential for misclassification, the exclusion of cancer patients solely based on PS assessment is unjustified. Designing clinical trials that produce broadly applicable evidence is both a clinical and an ethical imperative.
