# Supplementary material for: Impact of ECOG performance status 2 participants on outcomes of pivotal cancer clinical trials: a meta-analysis and meta-regression
Source: ESMO Open. 2026 Feb 2;11(2):106065. doi: 10.1016/j.esmoop.2026.106065 (PMC12887880; doi:10.1016/j.esmoop.2026.106065)
Supplement: Supplementary Data [file mmc1.docx]

**SUPPLEMENTARY MATERIALS**

1. **Study design**

**Table S1** PRISMA 2020 checklist.

**Figure S1** PRISMA flow diagram of search and study selection process.

**Table S2** Definition of the outcome "AE-related deaths".

**Table S3** Performance status scale conversion.

**Table S4** Main characteristics of the included clinical trials.

**Table S5** Summary of included clinical trials’ characteristics.

**Table S6** Pooled HRs for PFS and OS of clinical trials reporting PS 0 and PS 1 separately.

1. **Risk of bias assessment**

**Figure S2** Summary plot.

**Figure S3** Traffic lights plot.

1. **Publication bias**

**Figure S4** Funnel plot of HRs for PFS.

**Figure S5** Funnel plot of HRs for OS.

**Figure S6** Funnel plot of proportions for any-grade AEs.

**Figure S7** Funnel plot of proportions for high-grade AEs.

**Figure S8** Funnel plot of proportions for SAEs.

**Figure S9** Funnel plot of proportions for dose interruptions.

**Figure S10** Funnel plot of proportions for dose reductions.

**Figure S11** Funnel plot of proportions for dose discontinuations.

**Figure S12** Funnel plot of proportions for AE-related deaths.

1. **Efficacy outcomes**

**Figure S13** Subgroup analysis of PFS according to PS and tumor type.

**Figure S14** Subgroup analysis of OS according to PS and tumor type.

**Figure S15** Subgroup analysis of PFS according to PS and drug class.

**Figure S16** Subgroup analysis of OS according to PS and drug class.

**Figure S17** Forest plot of pooled HRs for PFS.

**Table S7** Meta-regression model for PFS including the “total number of study participants”.

**Table S8** Meta-regression models for PFS using arcsine-transformed proportions.

**Figure S18** Forest plot of pooled HRs for OS.

**Table S9** Meta-regression models for OS using arcsine-transformed proportions.

**Table S10** Meta-regression model for OS including the “total number of study participants”.

1. **Safety outcomes**

**Figure S19** Forest plot of pooled proportions for any-grade AEs.

**Table S11** Meta-regression models for any-grade AEs using arcsine-transformed proportions.

**Figure S20** Forest plot of pooled proportions for high-grade AEs.

**Table S12** Meta-regression models for high-grade AEs using arcsine-transformed proportions.

**Figure S21** Forest plot of pooled proportions for SAEs.

**Table S13** Meta-regression models for SAEs using arcsine-transformed proportions.

**Figure S22** Forest plot of pooled proportions for dose interruptions.

**Table S14** Meta-regression models for dose interruptions using arcsine-transformed proportions.

**Figure S23** Forest plot of pooled proportions for dose reductions.

**Table S15** Meta-regression models for dose reductions using arcsine-transformed proportions.

**Figure S24** Forest plot of pooled proportions for dose discontinuations.

**Table S16** Meta-regression models for dose discontinuations using arcsine-transformed proportions.

**Figure S25** Forest plot of pooled proportions for AE-related deaths.

**Table S17** Meta-regression models of AE-related deaths using arcsine-transformed proportions.

1. **Analyses of multicollinearity**

**Table S18** Variance inflation factors for covariates included in meta-regression models for both efficacy and safety outcomes.

1. **R packages used**
2. **List of abbreviations**
3. **References**
4. **STUDY DESIGN**

**Table S1 -** PRISMA 2020 checklist.

| **Section and Topic** | **Item #** | **Checklist item** |
| --- | --- | --- |
| **TITLE** | | |
| Title | 1 | Identify the report as a systematic review. |
| **ABSTRACT** | | |
| Abstract | 2 | See the PRISMA 2020 for Abstracts checklist. |
| **INTRODUCTION** | | |
| Rationale | 3 | Describe the rationale for the review in the context of existing knowledge. |
| Objectives | 4 | Provide an explicit statement of the objective(s) or question(s) the review addresses. |
| **METHODS** | | |
| Eligibility criteria | 5 | Specify the inclusion and exclusion criteria for the review and how studies were grouped for the syntheses. |
| Information sources | 6 | Specify all databases, registers, websites, organisations, reference lists and other sources searched or consulted to identify studies. Specify the date when each source was last searched or consulted. |
| Search strategy | 7 | Present the full search strategies for all databases, registers and websites, including any filters and limits used. |
| Selection process | 8 | Specify the methods used to decide whether a study met the inclusion criteria of the review, including how many reviewers screened each record and each report retrieved, whether they worked independently, and if applicable, details of automation tools used in the process. |
| Data collection process | 9 | Specify the methods used to collect data from reports, including how many reviewers collected data from each report, whether they worked independently, any processes for obtaining or confirming data from study investigators, and if applicable, details of automation tools used in the process. |
| Data items | 10a | List and define all outcomes for which data were sought. Specify whether all results that were compatible with each outcome domain in each study were sought (e.g. for all measures, time points, analyses), and if not, the methods used to decide which results to collect. |
|  | 10b | List and define all other variables for which data were sought (e.g. participant and intervention characteristics, funding sources). Describe any assumptions made about any missing or unclear information. |
| Study risk of bias assessment | 11 | Specify the methods used to assess risk of bias in the included studies, including details of the tool(s) used, how many reviewers assessed each study and whether they worked independently, and if applicable, details of automation tools used in the process. |
| Effect measures | 12 | Specify for each outcome the effect measure(s) (e.g. risk ratio, mean difference) used in the synthesis or presentation of results. |
| Synthesis methods | 13a | Describe the processes used to decide which studies were eligible for each synthesis (e.g. tabulating the study intervention characteristics and comparing against the planned groups for each synthesis (item #5)). |
|  | 13b | Describe any methods required to prepare the data for presentation or synthesis, such as handling of missing summary statistics, or data conversions. |
|  | 13c | Describe any methods used to tabulate or visually display results of individual studies and syntheses. |
|  | 13d | Describe any methods used to synthesize results and provide a rationale for the choice(s). If meta-analysis was performed, describe the model(s), method(s) to identify the presence and extent of statistical heterogeneity, and software package(s) used. |
|  | 13e | Describe any methods used to explore possible causes of heterogeneity among study results (e.g. subgroup analysis, meta-regression). |
|  | 13f | Describe any sensitivity analyses conducted to assess robustness of the synthesized results. |
| Reporting bias assessment | 14 | Describe any methods used to assess risk of bias due to missing results in a synthesis (arising from reporting biases). |
| Certainty assessment | 15 | Describe any methods used to assess certainty (or confidence) in the body of evidence for an outcome. |
| **RESULTS** | | |
| Study selection | 16a | Describe the results of the search and selection process, from the number of records identified in the search to the number of studies included in the review, ideally using a flow diagram. |
|  | 16b | Cite studies that might appear to meet the inclusion criteria, but which were excluded, and explain why they were excluded. |
| Study characteristics | 17 | Cite each included study and present its characteristics. |
| Risk of bias in studies | 18 | Present assessments of risk of bias for each included study. |
| Results of individual studies | 19 | For all outcomes, present, for each study: (a) summary statistics for each group (where appropriate) and (b) an effect estimate and its precision (e.g. confidence/credible interval), ideally using structured tables or plots. |
| Results of syntheses | 20a | For each synthesis, briefly summarise the characteristics and risk of bias among contributing studies. |
|  | 20b | Present results of all statistical syntheses conducted. If meta-analysis was done, present for each the summary estimate and its precision (e.g. confidence/credible interval) and measures of statistical heterogeneity. If comparing groups, describe the direction of the effect. |
|  | 20c | Present results of all investigations of possible causes of heterogeneity among study results. |
|  | 20d | Present results of all sensitivity analyses conducted to assess the robustness of the synthesized results. |
| Reporting biases | 21 | Present assessments of risk of bias due to missing results (arising from reporting biases) for each synthesis assessed. |
| Certainty of evidence | 22 | Present assessments of certainty (or confidence) in the body of evidence for each outcome assessed. |
| **DISCUSSION** | | |
| Discussion | 23a | Provide a general interpretation of the results in the context of other evidence. |
|  | 23b | Discuss any limitations of the evidence included in the review. |
|  | 23c | Discuss any limitations of the review processes used. |
|  | 23d | Discuss implications of the results for practice, policy, and future research. |
| **OTHER INFORMATION** | | |
| Registration and protocol | 24a | Provide registration information for the review, including register name and registration number, or state that the review was not registered. |
|  | 24b | Indicate where the review protocol can be accessed, or state that a protocol was not prepared. |
|  | 24c | Describe and explain any amendments to information provided at registration or in the protocol. |
| Support | 25 | Describe sources of financial or non-financial support for the review, and the role of the funders or sponsors in the review. |
| Competing interests | 26 | Declare any competing interests of review authors. |
| Availability of data, code and other materials | 27 | Report which of the following are publicly available and where they can be found: template data collection forms; data extracted from included studies; data used for all analyses; analytic code; any other materials used in the review. |

**Figure S1** - PRISMA flow diagram of search and study selection process.

**
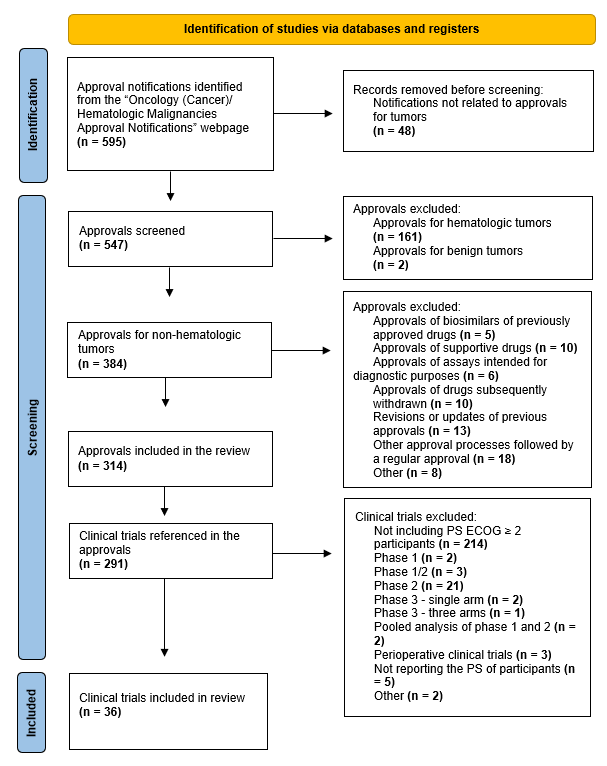
**

**Table S2 -** Definition of “AE-related deaths"

|  | Due to variability in reporting among included clinical trials, we applied the following hierarchy to extract the outcome “AE-related death”: |
| --- | --- |
| **1** | Fatal AEs were used when reported. |
| **2** | If fatal AEs were not reported but AEs leading to death were available, these were considered equivalent to fatal AEs. |
| **3** | If neither fatal AEs nor AEs leading to death were reported, treatment-related deaths were used. |
| **4** | If none of the above were available, all-cause deaths occurring on study were used. |

**Table S3** - Performance status scale conversion*

|  | **ECOG/WHO PS/ GOG PS** |  | **Karnofsky PS** |
| --- | --- | --- | --- |
| 0 | Fully active, able to carry on all pre-disease performance without restriction | 100 | Normal, no complaints, no evidence of disease |
|  |  | 90 | Able to carry on normal activity; minor signs or symptoms of disease |
| 1 | Restricted in physically strenuous activity but ambulatory and able to carry out work of a light or sedentary nature, e.g., light housework, office work | 80 | Normal activity with effort; some signs or symptoms of disease |
|  |  | 70 | Cares for self, unable to carry on  normal activity or to do active work |
| 2 | Ambulatory and capable of all selfcare but unable to carry out any work activities; up and about more than 50% of waking hours | 60 | Requires occasional assistance but  is able to care for most of personal  needs |
|  |  | 50 | Requires considerable assistance  and frequent medical care |
| 3 | Capable of only limited selfcare; confined to bed or chair more than 50% of waking hours | 40 | Disabled; requires special care and  assistance |
|  |  | 30 | Severely disabled; hospitalization is  indicated. Death not imminent |
| 4 | Completely disabled; cannot carry on any selfcare; totally confined to bed or chair | 20 | Very ill; hospitalization and active supportive care necessary |
|  |  | 10 | Moribund |
| 5 | Dead | 0 | Dead |

*Reproduced from “Cheng S, Qureshi M, Pullenayegum E, Haynes A, Chan KK. Do patients with reduced or excellent performance status derive the same clinical benefit from novel systemic cancer therapies? A systematic review and meta-analysis. ESMO Open. 2017 Sep 21;2(4):e000225. doi: 10.1136/esmoopen-2017-000225. PMID: 29209535”.

**Table S4** - Main characteristics of the included clinical trials.

**[A]**

| **Date of Approval** | **Clinical Trial (NCT)** | **First Author** | **Journal** | **Tumor Type** | **Experimental Arm** | **Control Arm** | **Primary Endpoint(s)** | **Eligible PS** |
| --- | --- | --- | --- | --- | --- | --- | --- | --- |
| 18-Dec-24 | NCT02767804 ^1^ | Horn L | Jama Oncol. | NSCLC | Ensartinib 225 mg PO daily | Crizotinib 250 mg PO twice daily | PFS | ECOG PS 0-2 |
| 27-Sep-24 | NCT04211337 ^2^ | Hadoux J | N Engl J Med. | TC | Selpercatinib 160 mg PO twice daily | Cabozantinib 140 mg PO daily OR Vandetanib 300 mg PO daily | PFS | ECOG PS 0-2 |
| 17-Jun-24 | NCT03914612* ^3^ | Eskander RN | N Engl J Med. | EC | Paclitaxel 175mg/m2 IV + Carboplatin AUC 5 IV + Pembrolizumab 200 mg IV on day 1 of a 21-day cycle (6 cycles) followed by Pembrolizumab maintenance 400 mg (up to 14 cycles) | Paclitaxel 175mg/m2 IV + Carboplatin AUC 5 IV + Placebo 200 mg IV (6 cycles) followed by Placebo maintenance 400 mg (up to 14 cycles) | PFS | ECOG PS 0-2 |
| 15-Dec-23 | NCT04223856 ^4^ | Powles T | N Engl J Med. | BLC | Enfortumab vedotin 1.25 mg/Kg IV on days 1 and 8 + Pembrolizumab 200 mg IV on day 1 of a 21-day cycle | Gemcitabine 1000 mg/m2 IV on days 1 and 8 + Cisplatin 70 mg/m2 IV on day 1 OR Carboplatin AUC 4.5 to 5 IV on day 1 of a 21-day cycle | PFS, OS | ECOG PS 0-2 |
| 27-Nov-23 | NCT03785964 ^5^ | Gounder M | N Engl J Med. | STS | Nirogacestat 150 mg PO daily | Placebo | PFS, OS | ECOG PS 0-2 |
| 23-Mar-22 | NCT03511664 ^6^ | Sartor O | N Engl J Med. | PCa | ^177^Lu-PSMA-617 plus protocol- standard care | Standard care alone (control group) | ibPFS, OS | ECOG PS 0-2 |
| 25-Jan-22 | NCT03070392 ^7^ | Nathan P | N Engl J Med. | Melanoma | Tebentafusp IV at a dose of 20 μg on day 1, 30 μg on day 8, and 68 μg weekly thereafter | Pembrolizumab 2mg/Kg or 200 mg IV on day 1 of a 21-day cycle OR Ipilimumab 3mg/Kg IV on day 1 of a 21-day cycle (maximum of 4 doses) OR Dacarbazine 1000 mg/m2 IV on day 1 of a 21-day cycle | OS | ECOG PS 0-2 |
| 3-Mar-21 | NCT03052608 ^8^ | Shaw AT | N Engl J Med. | NSCLC | Lorlatinib 100 mg PO daily | Crizotinib 250 mg PO twice daily | PFS | ECOG PS 0-2 |
| 22-May-20 | NCT02737501 ^9^ | Camidge DR | J Clin Oncol. | NSCLC | Brigatinib 180 mg PO daily | Crizotinib 250 mg PO twice daily | PFS | ECOG PS 0-2 |
| 19-May-20 | NCT02987543 ^10^ | de Bono J | N Engl J Med. | PCa | Olaparib 300 mg PO twice daily | Enzalutamide 160 mg PO once daily OR Abiraterone 1000 mg PO once daily + Prednisone 5 mg PO twice daily. | ibPFS | ECOG PS 0-2 |
| 15-May-20 | NCT03353753 ^11^ | Blay JY | Lancet Oncol. | GIST | Ripretinib 150 mg PO daily | Placebo | PFS | ECOG PS 0-2 |
| 7-Feb-18 | NCT01715285 ^12^ | Fizazi K | Lancet Oncol. | PCa | Abiraterone acetate 1000 mg PO daily + Prednisone 5 mg PO once daily + ADT | Placebo + ADT | OS, ibPFS | ECOG PS 0-2 |
| 6-Nov-17 | NCT02075840 ^13^ | Peters S | N Engl J Med. | NSCLC | Alectinib 600 mg PO twice daily | Crizotinib 250 mg PO twice daily | PFS | ECOG PS 0-2 |
| 26-May-17 | NCT01828099 ^14^ | Soria JC | Lancet. | NSCLC | Ceritinib 750 mg PO daily | Cisplatin 75 mg/m² IV on day 1 OR Carboplatin AUC 5–6 IV on day 1 + Pemetrexed 500 mg/m² on day 1 of a 21-day cycle (for 4 cycles) followed by maintenance Pemetrexed | PFS | WHO PS 0-2 |
| 18-May-17 | NCT02256436 ^15^ | Bellmunt J | N Engl J Med | BLC | Pembrolizumab 200 mg IV on day 1 of a 21-day cycle | Paclitaxel 175 mg/m2 IV on day 1 of a 21-day cycle OR Docetaxel 75 mg/m2 IV on day 1 of a 21-day cycle OR Vinflunine 320 mg/m2 IV on day 1 of a 21-day cycle | PFS, OS | ECOG PS 0-2 |
| 31-Mar-17 | NCT01740427 ^16^ | Slamon DJ | J Clin Oncol. | BrC | Palbociclib 125mg PO daily from day 1 to day 21 of a 28-day cycle + Letrozole 2,5mg PO daily | Letrozole 2,5mg PO daily | PFS | ECOG PS 0-2 |
| 27-Mar-17 | NCT01847274 ^17^ | Mirza MR | N Engl J Med. | OC | Niraparib 300 mg PO daily | Placebo | PFS | ECOG PS 0-2 |
| 28-Jan-16 | NCT01327885 ^18^ | Schöffski P. | Lancet. | STS | Eribulin mesilate 1,4 mg/m² IV on days 1 and 8 of a 21-day cycle | Dacarbazine (850 mg/m², 1000 mg/m², or 1200 mg/m² IV on day 1 of a 21-day cycle. | OS | ECOG PS 0-2 |
| 24-Nov-15 | NCT00981058 ^19^ | Thatcher N | Lancet Oncol. | NSCLC | Gemcitabine 1250 mg/m² IV on days 1 and 8 + Cisplatin 75 mg/m² IV on day 1 of a 21-day cycle (up to 6 cycles) + Necitumumab 800 mg IV on days 1 and 8 of a 21-day cycle | Gemcitabine 1250 mg/m² IV on days 1 and 8 + Cisplatin 75 mg/m² IV on day 1 of a 21-day cycle (up to 6 cycles) | OS | ECOG PS 0-2 |
| 13-Jul-15 | NCT00322452** ^20^ | Yi-Long Wu | Lung Cancer | NSCLC | Gefitinib 250 mg PO daily | Paclitaxel IV on Day 1 + Carboplatin AUC 5.0 or 6.0 IV on Day 1 of a 21-day cycle (for ≤ 6 cycles) | PFS | ECOG PS 0-2 |
| 13-Feb-15 | NCT01321554 ^21^ | Schlumberger M | N Engl J Med. | TC | Lenvatinib 24 mg PO daily | Placebo | PFS | ECOG PS 0-2 |
| 14-Nov-14 | NCT00976911 ^22^ | Husain A | Gynecol Oncol. | OC | Paclitaxel 80 mg/m2 once weekly; topotecan 4 mg/m2 on days 1, 8, and 15 every 4 weeks or 1.25 mg/kg on days 1–5 every 3 weeks; or pegylated liposomal doxorubicin 40 mg/m2 every 4 weeks + bevacizumab (10 mg/kg every 2 weeks) | Paclitaxel 80 mg/m2 once weekly; topotecan 4 mg/m2 on days 1, 8, and 15 every 4 weeks or 1.25 mg/kg on days 1–5 every 3 weeks; or pegylated liposomal doxorubicin 40 mg/m2 every 4 weeks | PFS | ECOG PS 0-2 |
| 22-Nov-13 | NCT00984282 ^23^ | Brose MS | Lancet. | TC | Sorafenib 400mg PO daily | Placebo | PFS | ECOG PS 0-2 |
| 20-Nov-13 | [NCT00932451](http://clinicaltrials.gov/show/NCT00932451) ^24^ | Shaw AT | N Engl J Med. | NSCLC | Crizotinib 250 mg PO twice daily | Pemetrexed 500 mg/m2 IV OR Docetaxel 75 mg/m2 of a 21-day cycle. | PFS | ECOG PS 0-2 |
| 15-May-13 | NCT00699751 ^25^ | Parker C | N Engl J Med. | PCa | Radium-223 50 kBq/Kg IV | Placebo | OS | ECOG PS 0-2 |
| 14-May-13 | NCT00446225 ^26^ | Rosell R | Lancet Oncol. | NSCLC | Erlotinib 150 mg PO daily | Cisplatin 75 mg/m² IV on day 1 + Docetaxel 75 mg/m² IV on day 1 OR Gemcitabine 1250 mg/m² IV on days 1 and 8 of a 21-day cycle. | PFS | ECOG PS 0-2 |
| 23-Jan-13 | NCT00700102 ^27^ | Bennouna J | Lancet Oncol. | CRC | Fluorouracil or oral capecitabine + Irinotecan OR Oxaliplatin with Bevacizumab at 2,5 mg/kg per week equivalent | Fluorouracil or oral capecitabine + Irinotecan OR Oxaliplatin | OS | ECOG PS 0-2 |
| 31-Aug-12 | NCT00974311 ^28^ | Scher HI | N Engl J Med. | PCa | Enzalutamide 160mg PO daily | Placebo | OS | ECOG PS 0-2 |
| 3-Aug-12 | NCT00561470 ^29^ | Van Cutsem E. | J Clin Oncol. | CRC | Aflibercept + FOLFIRI (Irinotecan 180mg/m2 IV, Leucovorin 400 mg/m2 IV, followed by Fluorouracil 400 mg/m2 bolus and Fluorouracil 2400 mg/m2 continuous infusion over 46 hours) | Placebo + FOLFIRI (Irinotecan 180mg/m2 IV, Leucovorin 400 mg/m2 IV, followed by Fluorouracil 400 mg/m2 bolus and Fluorouracil 2400 mg/m2 continuous infusion over 46 hours) | OS | ECOG PS 0-2 |
| 6-Jul-12 | NCT00154102 ^30^ | Van Cutsem E. | N Engl J Med. | CRC | FOLFIRI (Irinotecan 180mg/m2 IV, Leucovorin 400 mg/m2 IV, followed by Fluorouracil 400 mg/m2 bolus and Fluorouracil 2400 mg/m2 continuous infusion over 46 hours) + Cetuximab 400 mg/m2 followed by 250 mg/m2 | FOLFIRI (Irinotecan 180mg/m2 IV, Leucovorin 400 mg/m2 IV, followed by Fluorouracil 400 mg/m2 bolus and Fluorouracil 2400 mg/m2 continuous infusion over 46 hours) + Placebo | PFS | ECOG PS 0-2 |
| 5-May-11 | NCT00510068 ^31^ | Yao JC | N Engl J Med. | NT | Everolimus 10 mg PO daily | Placebo | PFS | WHO PS 0-2 |
| 28-Apr-11 | [NCT00638690](http://clinicaltrials.gov/show/NCT00638690) ^32^ | de Bono JS | N Engl J Med. | PCa | Abiraterone acetate 1000mg PO daily + Prednisone 5 mg PO twice daily | Placebo | OS | ECOG PS 0-2 |
| 6-Apr-11 | [NCT00410761](http://clinicaltrials.gov/show/NCT00410761) ^33^ | Wells SA Jr. | J Clin Oncol. | TC | Vandetanib 300 mg PO daily | Placebo | PFS | WHO PS 0-2 |
| 15-Nov-10 | NCT00388726 ^34^ | Cortes J | Lancet. | BrC | Eribulin mesilate 1,4 mg/m² IV on days 1 and 8 of a 21-day cycle | Treatment of physician’s choice | OS | ECOG PS 0-2 |
| 20-Oct-10 | [NCT01041404](http://clinicaltrials.gov/show/NCT01041404) ^35^ | Bang YJ | Lancet. | GC | Trastuzumab + Chemotherapy (Capecitabine + Cisplatin OR Fluorouracil + Cisplatin) | Chemotherapy (Capecitabine + Cisplatin OR Fluorouracil + Cisplatin) | OS | ECOG PS 0-2 |
| 17-Jun-10 | NCT00417079 ^36^ | De Bono JS | Lancet. | PCa | Prednisone 10mg PO daily + Cabazitaxel 25 mg/m2 IV on day 1 of a 21-day cycle | Prednisone 10mg PO daily + Mitoxantrone 12 mg/m2 IV | OS | ECOG PS 0-2 |

**[B]**

|  | **Enrolled Participants** | | | | | **Events** | |  |  |
| --- | --- | --- | --- | --- | --- | --- | --- | --- | --- |
| **Clinical Trial (NCT)** | **ECOG PS 0** | **ECOG PS 1** | **ECOG PS 0-1** | **ECOG PS 2** | **Unknown/Other ECOG PS** | **PFS** | **OS** | **Were survival outcomes for PS2 reported?** | **How were survival outcomes reported?** |
| NCT02767804 ^1^ | - | - | 276 | 14 | 0 | 119 | 62 | No | - |
| NCT04211337 ^2^ | 177 | 109 | - | 3 | 2 | 59 | - | No | - |
| NCT03914612 ^3^ | 145 | 74 | - | 6 | 0 | 307 | - | No | - |
|  | 394 | 176 | - | 18 | 0 |  |  | No | - |
| NCT04223856 ^4^ | 438 | 420 | - | 26 | 2 | 530 | 359 | No | - |
| NCT03785964 ^5^ | 103 | 38 | - | 1 | 0 | 49 | - | No | - |
| NCT03511664 ^6^ | - | - | 768 | 63 | 0 | 347 | 530 | Yes (PFS – OS) | ECOG PS 0-1 vs. ECOG PS 2 |
| NCT03070392 ^7^ | 277 | 80 | - | 1 | 20 | - | 150 | No | - |
| NCT03052608 ^8^ | 124 | 160 | - | 12 | 0 | 127 | 51 | No | - |
| NCT02737501 ^9^ | 107 | 154 | - | 14 | 0 | 150 | 70 | No | - |
| NCT02987543 ^10^ | 118 | 113 | - | 14 | 1 | 174 | 93 | Yes (PFS) | ECOG PS 0 vs. ECOG PS 1 vs. ECOG PS 2 |
| NCT03353753 ^11^ | 54 | 0 | - | 0 | 75 | 88 | 52 | No | - |
| NCT01715285 ^12^ | - | - | - | 40 | 1159 | - | 618 | Yes (OS) | ECOG PS 0-1 vs. ECOG PS 2 |
| NCT02075840 ^13^ | 97 | 186 | - | 20 | 0 | 164 | 75 | Yes (PFS) | ECOG PS 0 vs. ECOG PS 1 vs. ECOG PS 2 |
| NCT01828099 ^14^ | 139 | 212 | - | 24 | 1 | 202 | 107 | No | - |
| NCT02256436 ^15^ | 225 | 301 | - | 6 | 10 | 437 | 334 | Yes (OS) | ECOG PS 0-1 vs. ECOG PS 2 |
| NCT01740427 ^16^ | 359 | 295 | - | 12 | 0 | - | 405 | No | - |
| NCT01847274 ^17^ | 139 | 64 | - | 0 | 0 | 316 | - | No | - |
|  | 238 | 112 | - | 0 | 0 |  |  | No | - |
| NCT01327885 ^18^ | 201 | 235 | - | 16 | 0 | - | 174 | Yes (OS) | ECOG PS 0 vs. ECOG PS 1 vs. ECOG PS 2 |
| NCT00981058 ^19^ | 344 | 652 | - | 96 | 0 | 848 | 860 | Yes (PFS – OS) | ECOG PS 0 vs. ECOG PS 1 vs. ECOG PS 2 |
| NCT00322452 ^20^ | - | - | 175 | 11 | 0 | 124 | - | No | - |
| NCT01321554 ^21^ | - | - | 377 | 15 | 0 | 220 | - | No | - |
| NCT00976911 ^22^ | 206 | 127 | - | 23 | 5 | 251 | - | Yes (PFS) | ECOG PS 0 vs. ECOG PS 1 vs. ECOG PS 2 |
| NCT00984282 ^23^ | 259 | 143 | - | 13 | 4 | - | - | No | - |
| [NCT00932451](http://clinicaltrials.gov/show/NCT00932451) ^24^ | 137 | 179 | - | 30 | 1 | 227 | 96 | Yes (PFS) | ECOG PS 0-1 vs. ECOG PS 2 |
| NCT00699751 ^25^ | 243 | 558 | - | 118 | 2 | - | 314 | Yes (OS) | ECOG PS 0-1 vs. ECOG PS 2 |
| NCT00446225 ^26^ | 57 | 92 | - | 24 | 0 | - | - | Yes (PFS) | ECOG PS 0 vs. ECOG PS 1 vs. ECOG PS 2 |
| NCT00700102 ^27^ | 357 | 421 | - | 38 | 4 | - | - | No | - |
| NCT00974311 ^28^ | - | - | 1097 | 102 | 1097 | - | 520 | Yes (OS) | ECOG PS 0-1 vs. ECOG PS 2 |
| NCT00561470 ^29^ | 699 | 500 | - | 27 | 0 | 847 | 863 | Yes (PFS – OS) | ECOG PS 0 vs. ECOG PS 1 vs. ECOG PS 2 |
| NCT00154102 ^30^ | 648 | 506 | - | 42 | 2 | 620 | 828 | No | - |
| NCT00510068 ^31^ | 272 | 126 | - | 12 | 0 | 237 | - | No | - |
| [NCT00638690](http://clinicaltrials.gov/show/NCT00638690) ^32^ | - | - | 1068 | 127 | 1068 | - | 552 | Yes (OS) | ECOG PS 0-1 vs. ECOG PS 2 |
| [NCT00410761](http://clinicaltrials.gov/show/NCT00410761) ^33^ | 212 | 105 | - | 14 | 0 | 124 | 48 | No | - |
| NCT00388726 ^34^ | 320 | 370 | - | 61 | 11 | 521 | 422 | No | - |
| [NCT01041404](http://clinicaltrials.gov/show/NCT01041404) ^35^ | - | - | 497 | 57 | 0 | 461 | 349 | Yes (OS) | ECOG PS 0-1 vs. ECOG PS 2 |
| NCT00417079 ^36^ | - | - | 694 | 61 | 0 | - | 513 | Yes (OS) | ECOG PS 0-1 vs. ECOG PS 2 |

**[C]**

|  | **PFS** | | | | | **OS** | | | | |
| --- | --- | --- | --- | --- | --- | --- | --- | --- | --- | --- |
| **Clinical Trial (NCT)** | **Overall HR (95%CI)** | **PS 0 HR (95%CI)** | **PS 1 HR (95%CI)** | **PS 0-1 HR (95%CI)** | **PS 2 HR (95%CI)** | **Overall HR (95%CI)** | **PS 0 HR (95%CI)** | **PS 1 HR (95%CI)** | **PS 0-1 HR (95%CI)** | **PS 2 HR (95%CI)** |
| NCT02767804 ^1^ | 0.45 (0.3 - 0.66) | - | - | 0.44 (0.29 - 0.65) | - | 0.91 (0.54 - 1.54) | - | - | - | - |
| NCT04211337 ^2^ | 0.29 (0.17 - 0.49) | - | - | 0.3 (0.18 - 0.51) | - | 0.37 (0.15 - 0.95) | - | - | - | - |
| NCT03914612 ^3^ | 0.3 (0.19 - 0.48) | - | - | - | - | - | - | - | - | - |
|  | 0.54 (0.41 - 0.71) | - | - | - | - | - | - | - | - | - |
| NCT04223856 ^4^ | 0.45 (0.38 - 0.54) | - | - | - | - | 0.47 (0.38 - 0.58) | - | - | - | - |
| NCT03785964 ^5^ | 0.29 (0.15 - 0.55) | - | - | - | - | - | - | - | - | - |
| NCT03511664 ^6^ | 0.4 (0.29 - 0.57) | - | - | 0.43 (0.33 - 0.56) | 0.18 (0.08 - 0.38) | 0.62 (0.52 - 0.74) | - | - | 0.61 (0.5 - 0.74) | 0.63 (0.35 - 1.13) |
| NCT03070392 ^7^ | 0.73 (0.58 - 0.94) | - | - | - | - | 0.51 (0.37 - 0.71) | - | - | - | - |
| NCT03052608 ^8^ | 0.28 (0.19 - 0.41) | - | - | - | - | 0.72 (0.41 - 1.25) | - | - | - | - |
| NCT02737501 ^9^ | 0.49 (0.35 - 0.68) | - | - | - | - | 0.92 (0.57 - 1.47) | - | - | - | - |
| NCT02987543 ^10^ | 0.34 (0.25 - 0.47) | 0.57 (0.36 - 0.95) | 0.25 (0.16 - 0.4) | 0.37 (0.26 - 0.51) | 0.25 (0.07 - 1.13) | 0.64 (0.43 - 0.97) | - | - | - | - |
| NCT03353753 ^11^ | 0.15 (0.09 - 0.25) | - | - | - | - | 0.36 (0.21 - 0.62) | - | - | - | - |
| NCT01715285 ^12^ | - | - | - | - | - | 0.66 (0.57 - 0.78) | - | - | 0.64 (0.54 - 0.75) | 1.42 (0.65 - 3.08) |
| NCT02075840 ^13^ | 0.48 (0.35 - 0.66) | 0.4 (0.21 - 0.77) | 0.48 (0.32 - 0.71) | 0.46 (0.33 - 0.64) | 0.74 (0.25 - 2.15) | 0.76 (0.48 - 1.2) | - | - | - | - |
| NCT01828099 ^14^ | 0.55 (0.42 - 0.73) | - | - | - | - | 0.73 (0.5 - 1.08) | - | - | - | - |
| NCT02256436 ^15^ | 0.98 (0.81 - 1.19) | - | - | - | - | 0.73 (0.59 - 0.91) | - | - | 0.74 (0.59 - 0.92) | 0.43 (0.04 - 4.2) |
| NCT01740427 ^16^ | - | - | - | - | - | 0.96 (0.78 - 1.18) | - | - | - | - |
| NCT01847274 ^17^ | 0.27 (0.17 - 0.41) | - | - | - | - | - | - | - | - | - |
|  | 0.45 (0.34 - 0.61) | - | - | - | - | - | - | - | - | - |
| NCT01327885 ^18^ | 0.88 (0.71 - 1.09) | - | - | - | - | 0.77 (0.62 - 0.95) | 0.58 (0.41 - 0.82) | 1.11 (0.83 - 1.48) | 0.85 (0.68 - 1.06) | 3 (0.25 - 35.79) |
| NCT00981058 ^19^ | 0.85 (0.74 - 0.98) | 0.84 (0.66 - 1.08) | 0.86 (0.73 - 1.02) | 0.85 (0.74 - 0.98) | 0.79 (0.5 - 1.24) | 0.84 (0.74 - 0.96) | 0.82 (0.64 - 1.05) | 0.85 (0.72 - 1.01) | 0.84 (0.73 - 0.97) | 0.78 (0.51 - 1.21) |
| NCT00322452 ^20^ | 0.54 (0.38 - 0.79) | - | - | - | - | - | - | - | - | - |
| NCT01321554 ^21^ | 0.21 (0.14 - 0.31) | - | - | 0.69 (0.49 - 0.87) | - | 0.73 (0.5 - 1.07) | - | - | - | - |
| NCT00976911 ^22^ | 0.484 (0.37 - 0.632) | 0.44 (0.31 - 0.62) | 0.73 (0.48 - 1.11) | 0.54 (0.41 - 0.71) | 0.47 (0.2 - 1.13) | - | - | - | - | - |
| NCT00984282 ^23^ | 0.59 (0.45 - 0.76) | - | - | - | - | 0.8 (0.54 - 1.19) | - | - | - | - |
| [NCT00932451](http://clinicaltrials.gov/show/NCT00932451) ^24^ | 0.49 (0.37 - 0.64) | - | - | 0.48 (0.36 – 0.63) | 0.31 (0.12 – 0.83) | 1.02 (0.68 - 1.54) | - | - | - | - |
| NCT00699751 ^25^ | - | - | - | - | - | 0.7 (0.58 - 0.83) | - | - | 0.68 (0.56 - 0.82) | 0.82 (0.5 - 1.35) |
| NCT00446225 ^26^ | 0.37 (0.25 - 0.54) | 0.26 (0.12 - 0.59) | 0.37 (0.22 - 0.62) | 0.33 (0.22 - 0.51) | 0.48 (0.15 - 1.48) | 1.04 (0.65 - 1.68) | - | - | - | - |
| NCT00700102 ^27^ | 0.68 (0.59 - 0.78) | - | - | - | - | 0.81 (0.69 - 0.94) | - | - |  |  |
| NCT00974311 ^28^ | - | - | - | - | - | 0.63 (0.53 - 0.75) | - | - | 0.62 (0.52 - 0.75) | 0.65 (0.39 - 1.07) |
| NCT00561470 ^29^ | 0.758 (0.661 - 0.869) | 0.761 (0.633 - 0.913) | 0.749 (0.607 - 0.923) | 0.76 (0.66 - 0.87) | 0.618 (0.259 - 1.475) | 0.817 (0.713 - 0.937) | 0.768 (0.635 - 0.928) | 0.869 (0.71 - 1.063) | 0.81 (0.71 - 0.93) | 0.978 (0.43 - 2.221) |
| NCT00154102 ^30^ | 0.85 (0.72 - 0.99) | - | - | - | - | 0.93 (0.81 - 1.07) | - | - | - | - |
| NCT00510068 ^31^ | 0.35 (0.27 - 0.45) | - | - | - | - | 1.05 (0.71 - 1.55) | - | - | - | - |
| [NCT00638690](http://clinicaltrials.gov/show/NCT00638690) ^32^ | - | - | - | - | - | 0.66 (0.56 - 0.79) | - | - | 0.64 (0.53 - 0.78) | 0.81 (0.53 - 1.24) |
| [NCT00410761](http://clinicaltrials.gov/show/NCT00410761) ^33^ | 0.46 (0.31 - 0.69) | - | - | - | - | 0.89 (0.48 - 1.65) | - | - | - | - |
| NCT00388726 ^34^ | 0.87 (0.71 - 1.05) | - | - | - | - | 0.81 (0.66 - 0.99) | - | - | - | - |
| [NCT01041404](http://clinicaltrials.gov/show/NCT01041404) ^35^ | 0.71 (0.59 - 0.85) | - | - | - | - | 0.74 (0.6 - 0.91) | - | - | 0.71 (0.56 - 0.89) | 0.96 (0.51 - 1.79) |
| NCT00417079 ^36^ | 0.74 (0.64 - 0.86) | - | - | - | - | 0.7 (0.59 - 0.83) | - | - | 0.68 (0.57 - 0.82) | 0.81 (0.48 - 1.38) |

**[D]**

| **Clinical Trial (NCT)** | **AE (All) (Event)** | **High-grade (≥G3) AE (Event)** | **SAE (Event)** | **Interruption of therapy (Event)** | **Dose reduction (Event)** | **Treatment discontinuation (Event)** | **Death (Event)** |
| --- | --- | --- | --- | --- | --- | --- | --- |
| NCT02767804 ^1^ | 286 | 134 | - | - | 63 | 23 | 0 |
| NCT04211337 ^2^ | 282 | 176 | 68 | 167 | 132 | 35 | 10 |
| NCT03914612 ^3^ | 726 | 395 | - | - | - | - | 3 |
| NCT04223856 ^4^ | 841 | 547 | 389 | 528 | 343 | 234 | 8 |
| NCT03785964 ^5^ | 138 | 50 | 22 | - | - | 15 | 1 |
| NCT03511664 ^6^ | 689 | 357 | 249 | 127 | 40 | 100 | 50 |
| NCT03070392 ^7^ | 334 | 128 | 95 | 11 | - | - | 0 |
| NCT03052608 ^8^ | 289 | 187 | 90 | 140 | 53 | 23 | 14 |
| NCT02737501 ^9^ | 272 | 183 | - | - | 86 | 29 | 20 |
| NCT02987543 ^10^ | 358 | 179 | - | - | - | - | 18 |
| NCT03353753 ^11^ | - | - | 11 | 15 | 6 | 5 | 25 |
| NCT01715285 ^12^ | 1118 | 735 | 347 | - | - | 36 | 65 |
| NCT02075840 ^13^ | 293 | 139 | 87 | 67 | 55 | 36 | 12 |
| NCT01828099 ^14^ | 359 | 256 | - | - | - | - | 17 |
| NCT02256436 ^15^ | 392 | 166 | - | - | - | 43 | 8 |
| NCT01740427 ^16^ | 654 | 417 | - | - | - | - | - |
| NCT01847274 ^17^ | 538 | 341 | 137 | 262 | 270 | 58 | 0 |
| NCT01327885 ^18^ | 429 | 265 | - | 146 | 90 | 28 | 13 |
| NCT00981058 ^19^ | 1062 | 721 | 460 | - | - | 301 | 25 |
| NCT01321554 ^21^ | 332 | 211 | 160 | 239 | 238 | 40 | 26 |
| NCT00984282 ^23^ | 387 | - | 132 | 191 | 152 | 47 | 18 |
| [NCT00932451](http://clinicaltrials.gov/show/NCT00932451) ^24^ | 340 | 207 | 104 | - | - | - | 32 |
| NCT00699751 ^25^ | 848 | 527 | 462 | - | - | 161 | - |
| NCT00446225 ^26^ | 163 | 93 | 52 | - | 41 | 30 | 3 |
| NCT00700102 ^27^ | 797 | 490 | 266 | - | - | 99 | 45 |
| NCT00974311 ^28^ | 1175 | 574 | 422 | - | - | 100 | 37 |
| NCT00561470 ^29^ | 1198 | 888 | - | - | - | 236 | - |
| NCT00154102 ^30^ | 843 | - | - | - | - | - | - |
| NCT00510068 ^31^ | - | - | - | - | - | 31 | 16 |
| [NCT00638690](http://clinicaltrials.gov/show/NCT00638690) ^32^ | - | - | - | - | - | 241 | 138 |
| [NCT00410761](http://clinicaltrials.gov/show/NCT00410761) ^33^ | - | - | - | - | 84 | 31 | 7 |
| NCT00388726 ^34^ | 727 | - | 190 | - | - | 105 | 38 |
| [NCT01041404](http://clinicaltrials.gov/show/NCT01041404) ^35^ | 576 | 399 | 176 | - | - | - | 13 |
| NCT00417079 ^36^ | - | - | - | - | - | - | 27 |

**Table S5 -** Summary of included clinical trials’ characteristics.

| **Included Clinical Trials (n = 36)** | **n (%)** |
| --- | --- |
|  |  |
| **Type of therapy** |  |
| Monotherapy | 26 (72.2) |
| Combined treatment | 10 (27.8) |
|  |  |
| **Drugs group** |  |
| Chemotherapy (single agent) | 3 (8.3) |
| Radiopharmaceuticals | 2 (5.6) |
| Hormonotherapy | 3 (8.3) |
| Hormonotherapy + Targeted therapy | 1 (2.8) |
| Other | 1 (2.8) |
| Targeted therapy (single agent) | 17 (47.2) |
| Immunotherapy (single agent) | 2 (5.6) |
| Chemotherapy + Targeted therapy | 6 (16.7) |
| Chemotherapy + Immunotherapy | 1 (2.8) |
|  |  |
| **Tumor group** |  |
| GI Non-Colorectal Cancer | 1 (2.8) |
| Lung Cancer | 9 (25) |
| Soft Tissue Sarcoma | 3 (8.3) |
| Neuroendocrine Tumors | 1 (2.8) |
| Thyroid Cancer | 4 (11.1) |
| GI Colorectal Cancer | 3 (8.3) |
| GU Non-Prostate Cancer | 2 (5.6) |
| GU Prostate Cancer | 7 (19.4) |
| Breast Cancer | 2 (5.6) |
| Gynecological Tumors | 3 (8.3) |
| Melanoma | 1 (2.8) |
|  |  |
| **Eligible PS** |  |
| ECOG PS 0-2 | 33 (91.7) |
| WHO PS 0-2 | 3 (8.3) |

**Table S6 -** Pooled HRs for PFS and OS of clinical trials reporting PS 0 and PS 1 separately.

| **Outcome** | **Clinical Trial (NCT)** | **ECOG PS 0**  **HR (95% CI)** | **ECOG PS 1**  **HR (95% CI)** | **ECOG PS 0-1**  **HR (95% CI)** |
| --- | --- | --- | --- | --- |
| **PFS** | NCT02987543 ^10^ | 0.57 (0.36 – 0.95) | 0.25 (0.16 – 0.40) | **0.37**  **(0.26 – 0.51)** |
|  | NCT02075840 ^13^ | 0.40 (0.21 – 0.77) | 0.48 (0.32 – 0.71) | **0.46**  **(0.33 – 0.64)** |
|  | NCT00981058 ^19^ | 0.84 (0.66 – 1.08) | 0.86 (0.73 – 1.02) | **0.85**  **(0.74 – 0.98)** |
|  | NCT00976911 ^22^ | 0.44 (0.31 – 0.62) | 0.73 (0.48 – 1.11) | **0.54**  **(0.41 – 0.71)** |
|  | NCT00446225 ^26^ | 0.26 (0.12 – 0.59) | 0.37 (0.22 – 0.62) | **0.33**  **(0.22 - 0.51)** |
|  | NCT00561470 ^29^ | 0.76 (0.63 – 0.91) | 0.75 (0.61 – 0.92) | **0.76**  **(0.66 – 0.87)** |
| **OS** | NCT01327885 ^18^ | 0.58 (0.41 – 0.82) | 1.11 (0.83 – 1.48) | **0.85**  **(0.68 – 1.06)** |
|  | NCT00981058 ^19^ | 0.82 (0.64 – 1.05) | 0.85 (0.72 – 1.01) | **0.84**  **(0.73 – 0.97)** |
|  | NCT00561470 ^29^ | 0.77 (0.64 – 0.93) | 0.87 (0.71 – 1.06) | **0.81**  **(0.71 – 0.93)** |

1. **RISK OF BIAS ASSESSMENT**

**Figure S2 –** Summary plot.


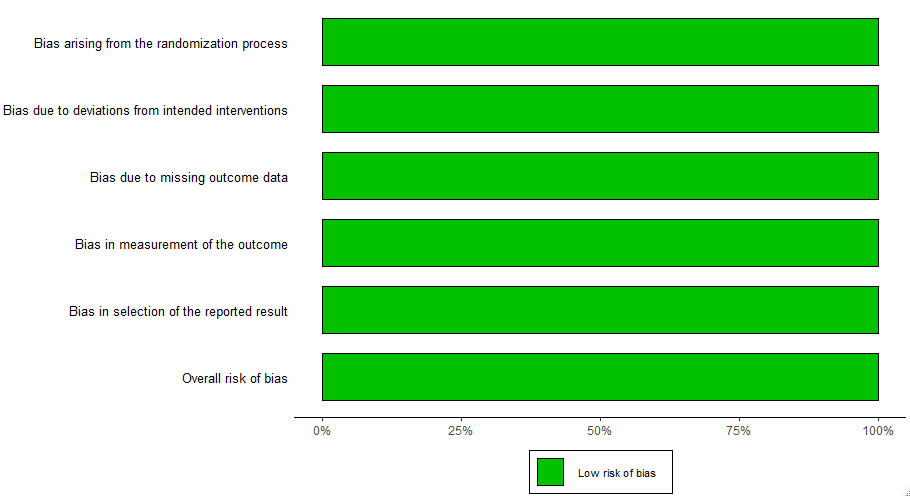


**Figure S3 –** Traffic lights plot.


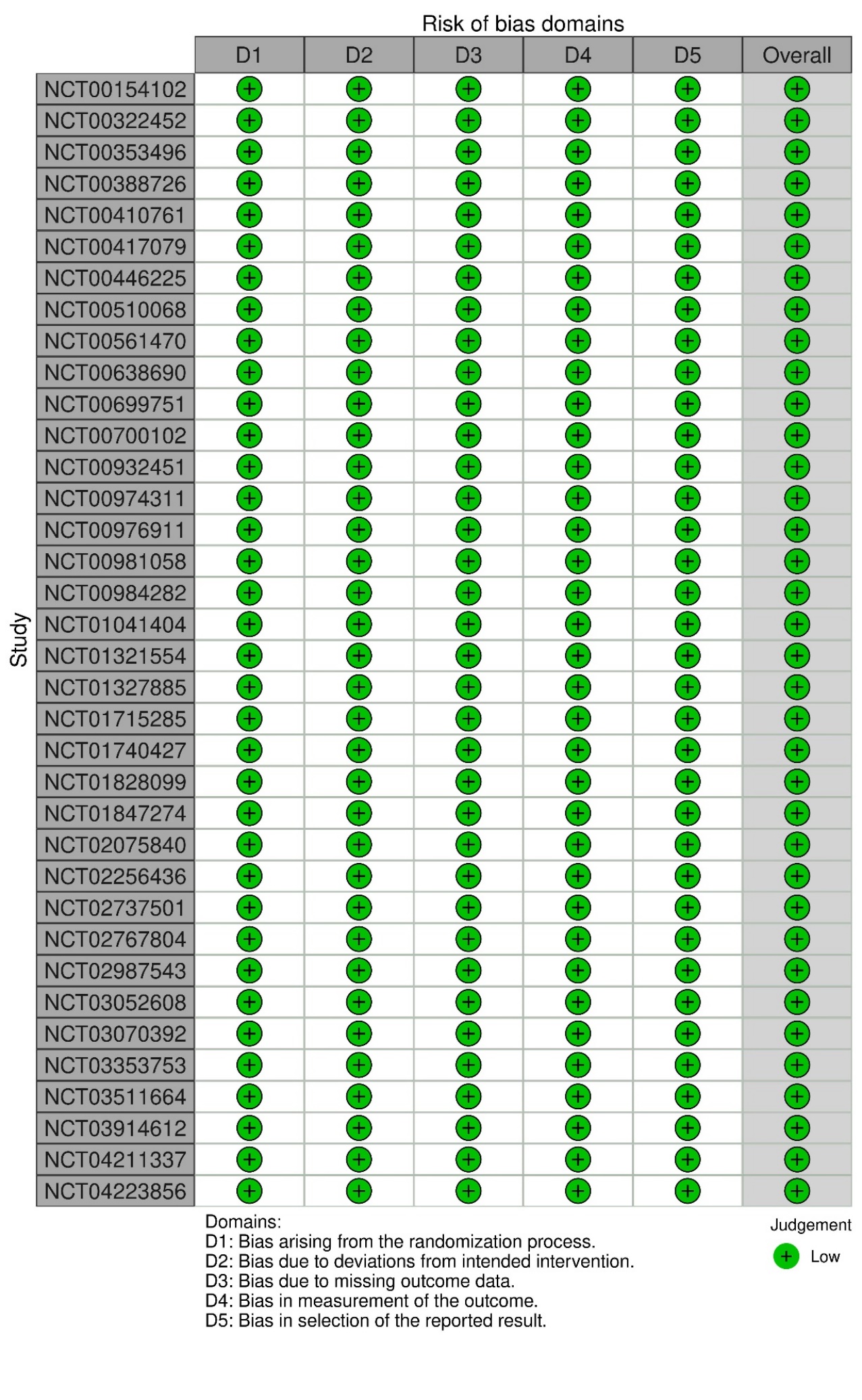


1. **PUBLICATION BIAS**

**Figure S4** - Funnel plot of HRs for PFS.


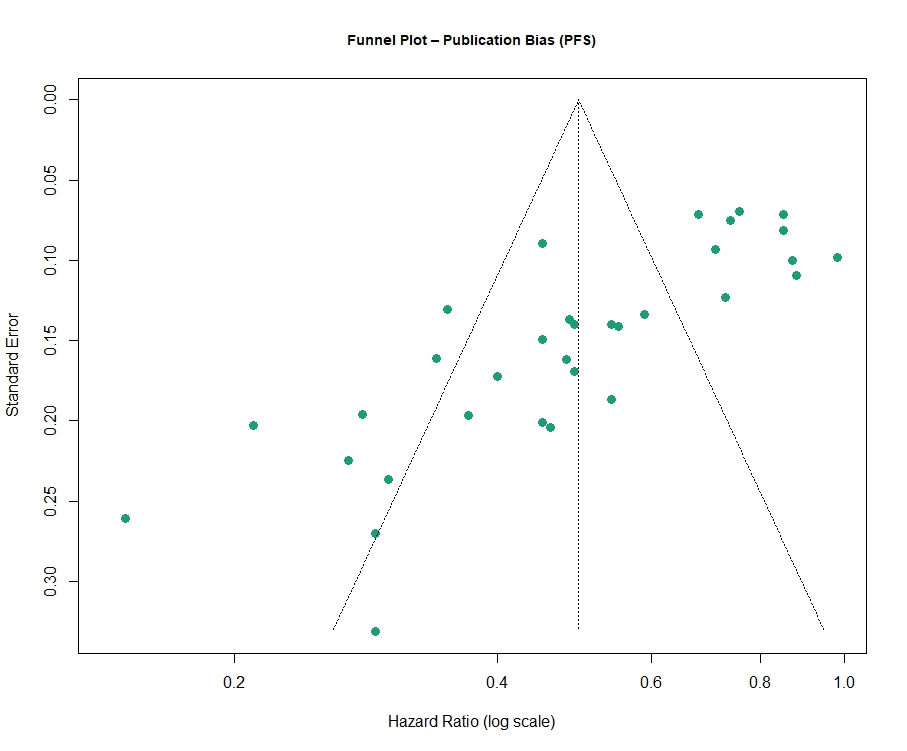


Egger’s test: p = <0.0001

**Figure S5** - Funnel plot of HRs for OS.


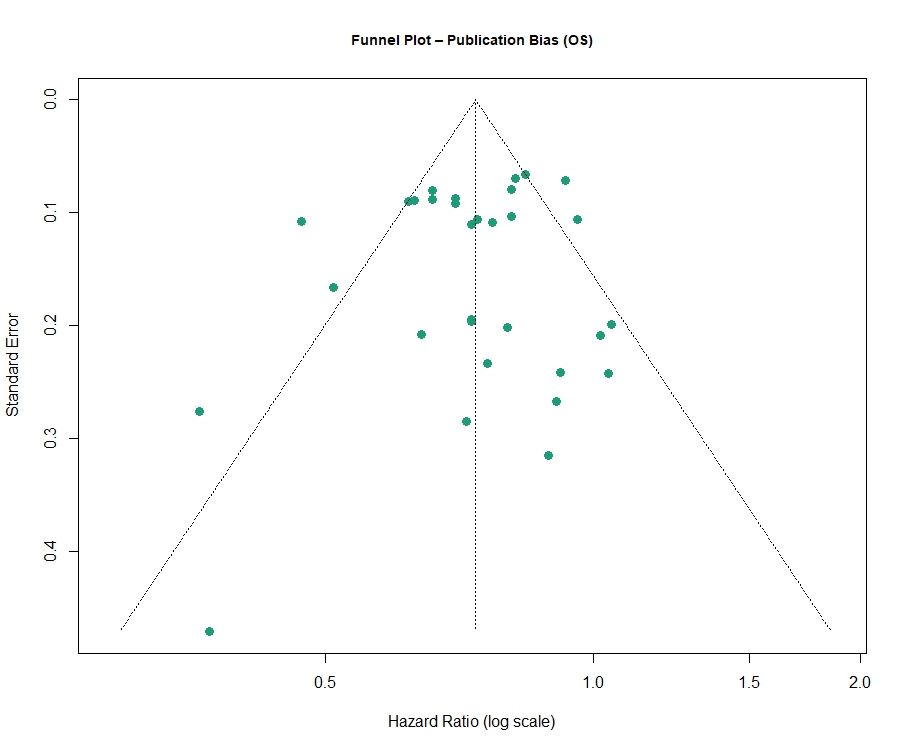


Egger’s test: p = 0.5675

**Figure S6** - Funnel plot of proportions for any-grade AEs.


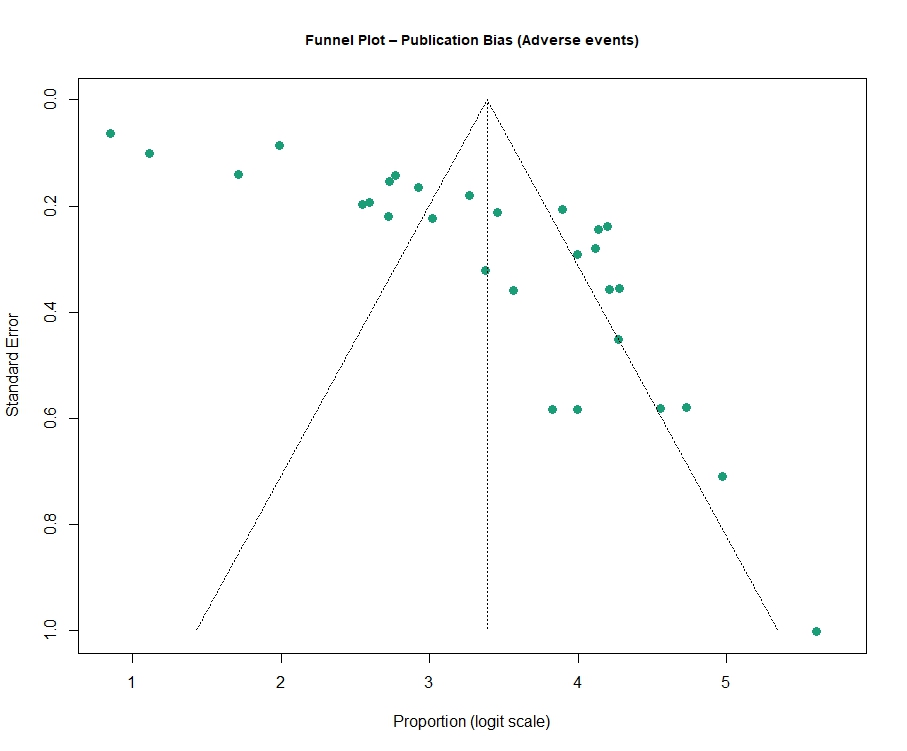


Egger’s test: p = <0.0001

**Figure S7** - Funnel plot of proportions for high-grade AEs.


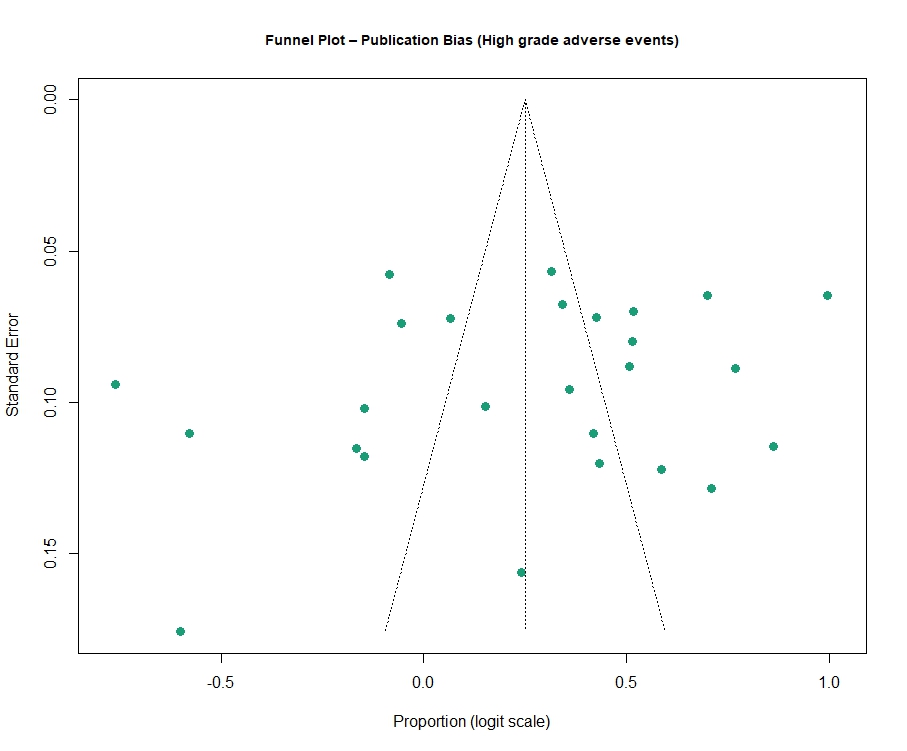


Egger’s test: p = 0.3134

**Figure S8** - Funnel plot of proportions for SAEs.


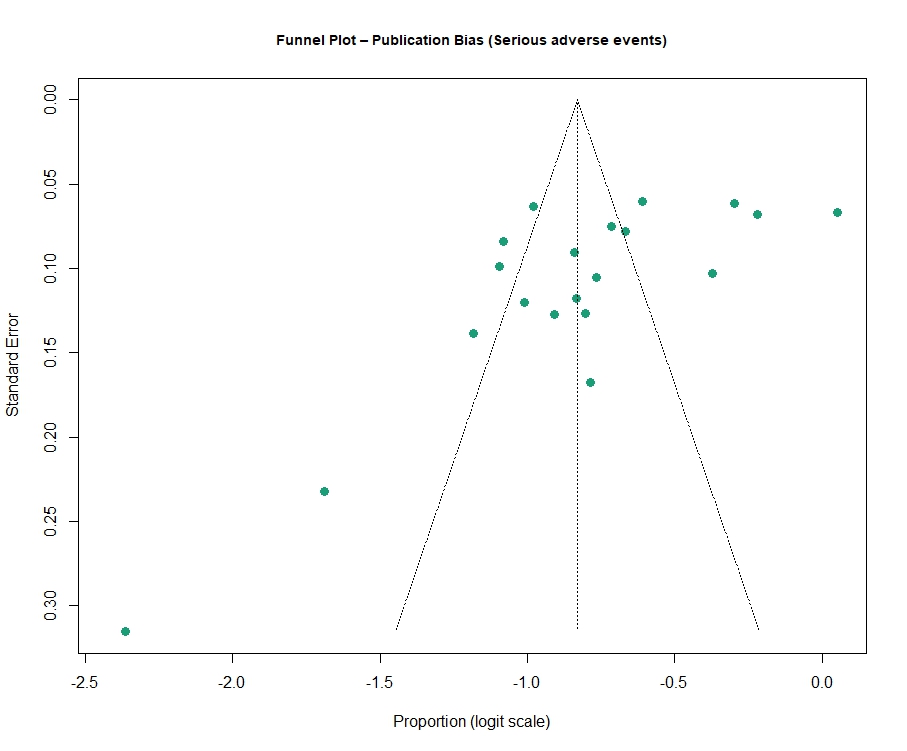


Egger’s test: p = 0.0094

**Figure S9** - Funnel plot of proportions for dose interruptions.


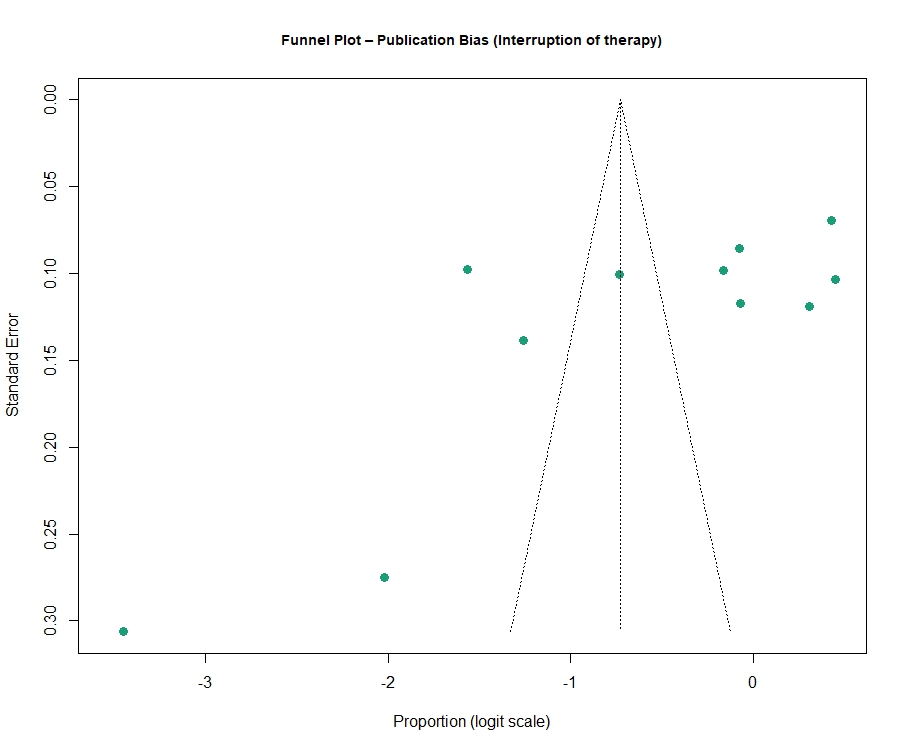


Egger’s test: p = 0.0426

**Figure S10** - Funnel plot of proportions for dose reductions.


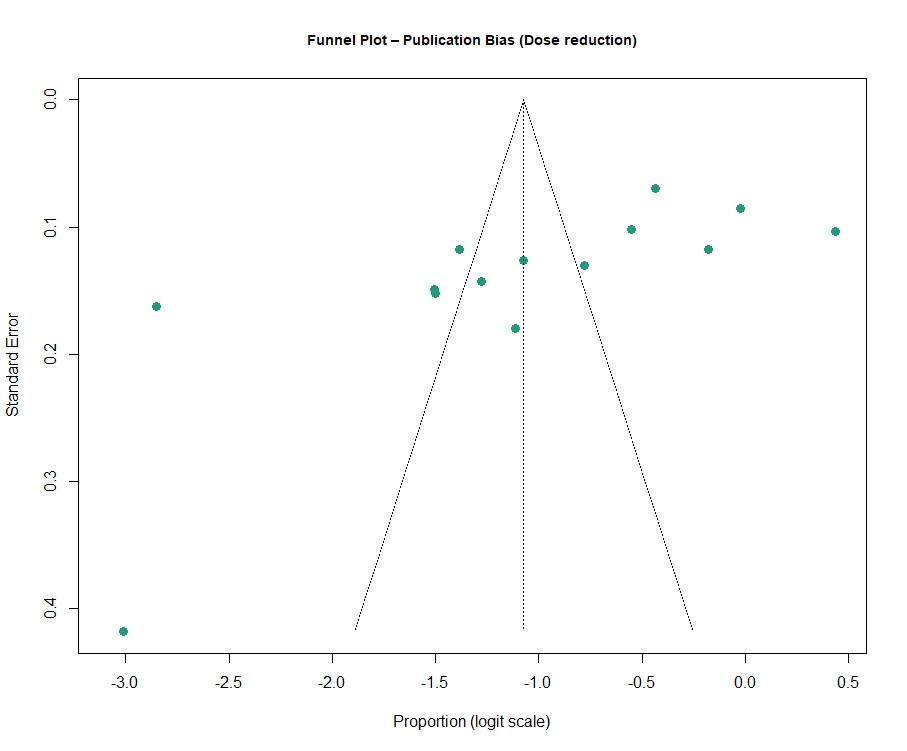


Egger’s test: p = 0.0093

**Figure S11** - Funnel plot of proportions for dose discontinuations.


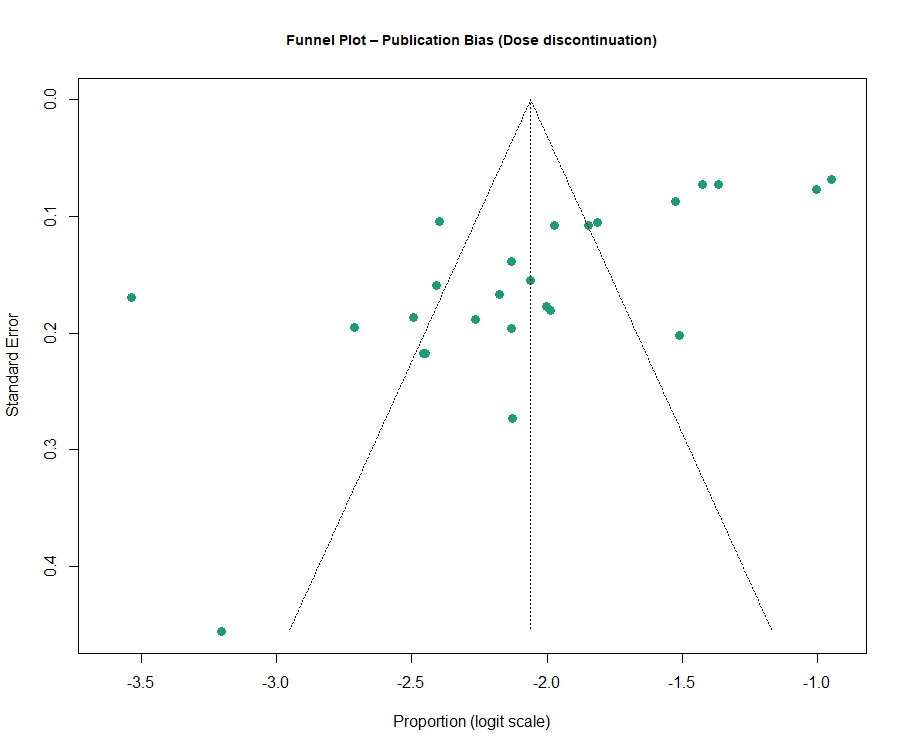


Egger’s test: p = <0.0001

**Figure S12** - Funnel plot of proportions for AE-related deaths.


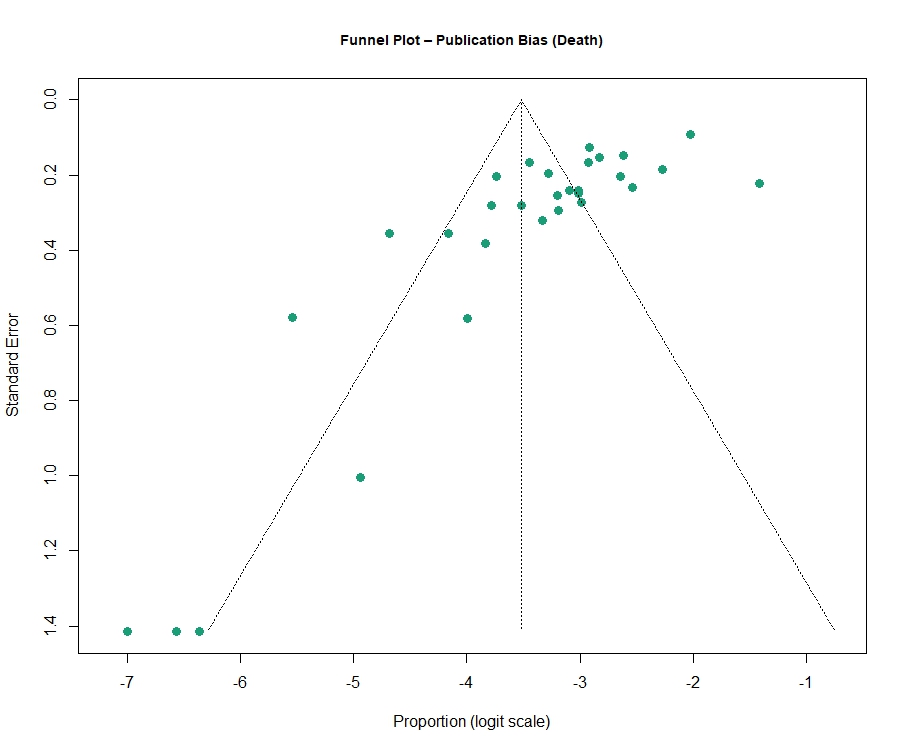


Egger’s test: p = <0.0001

1. **EFFICACY OUTCOMES**

**Figure S13 -** Subgroup analysis of PFS according to PS and tumor type.


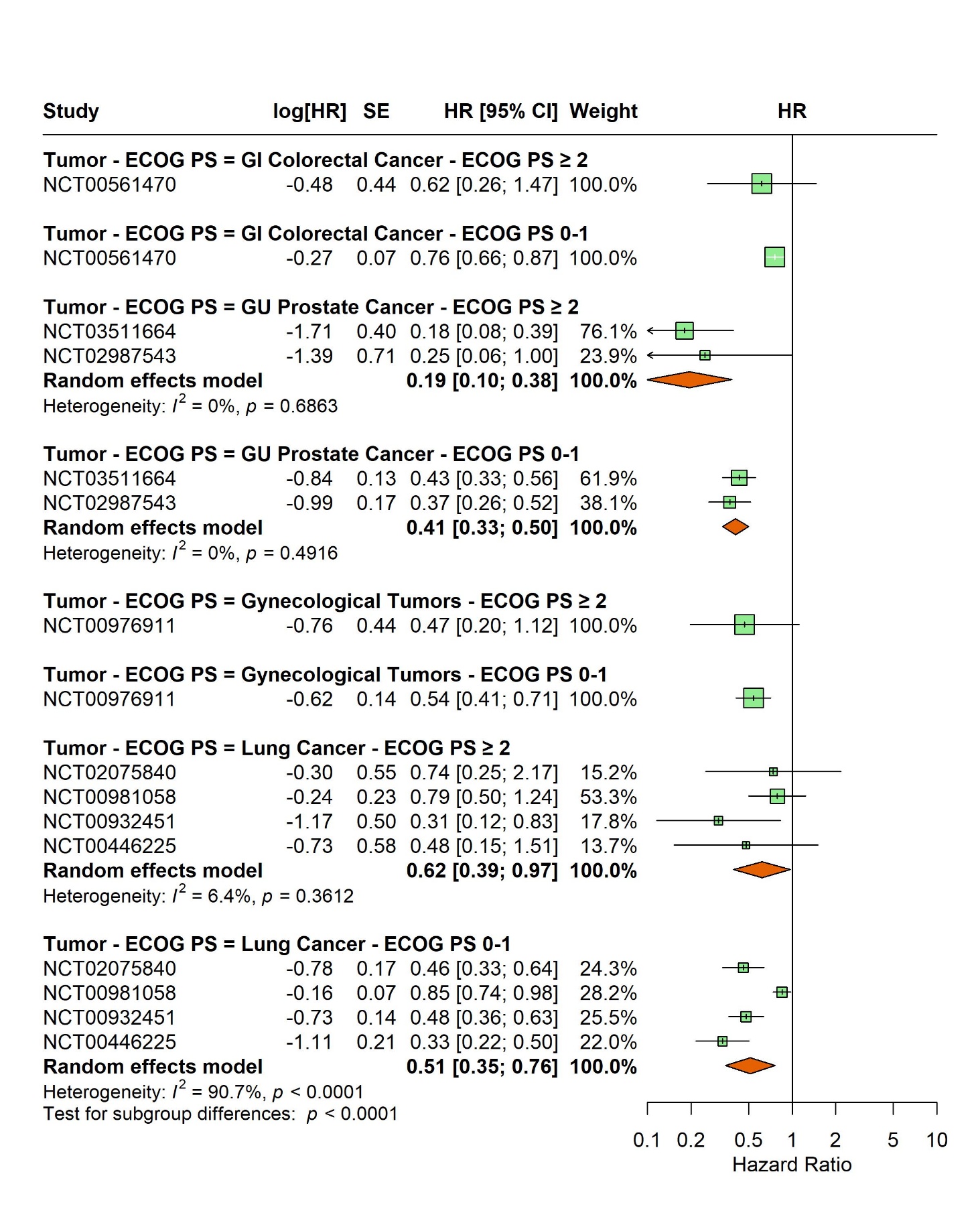


**Figure S14 -** Subgroup analysis of OS according to PS and tumor type.


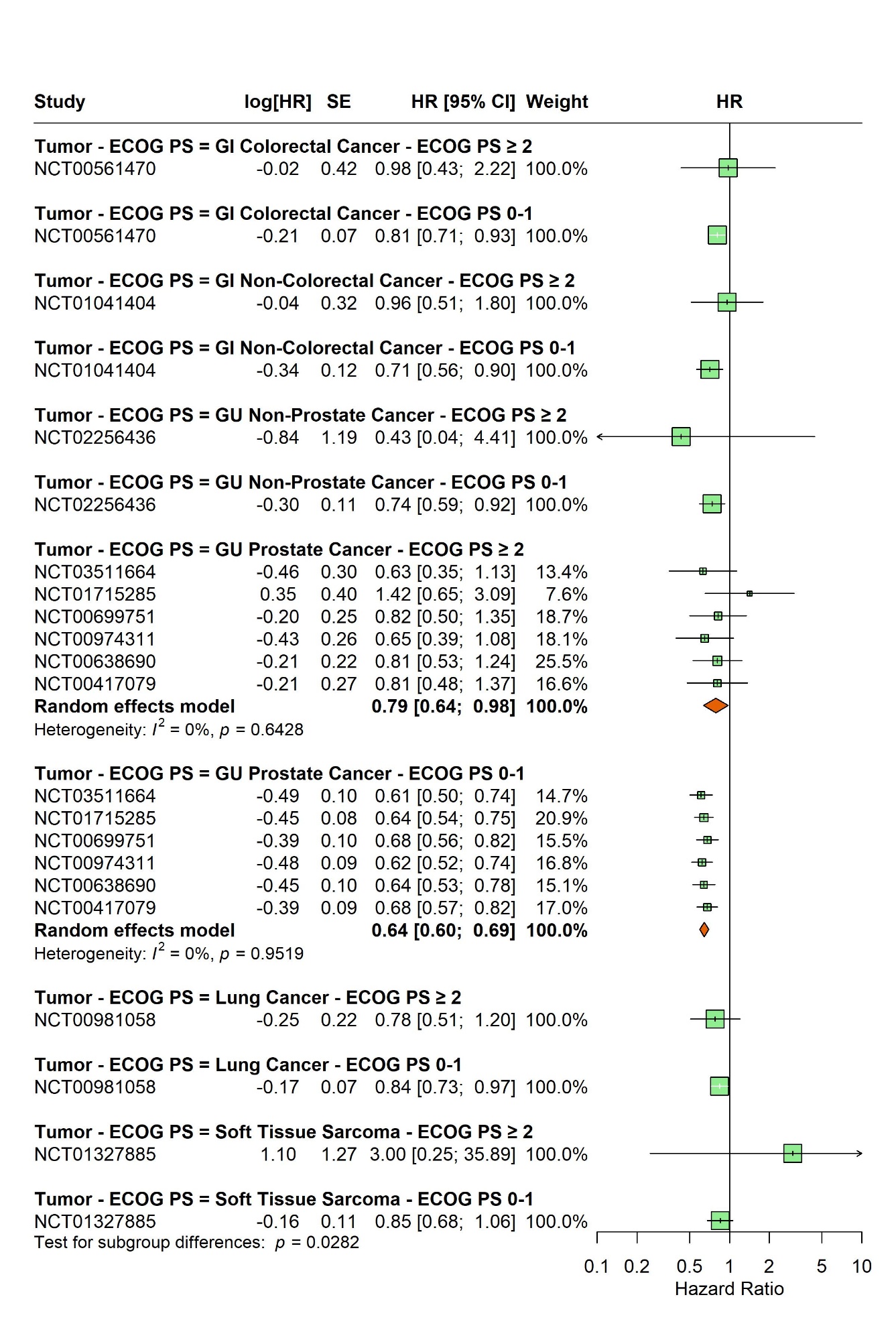


**Figure S15 -** Subgroup analysis of PFS according to PS and drug class.


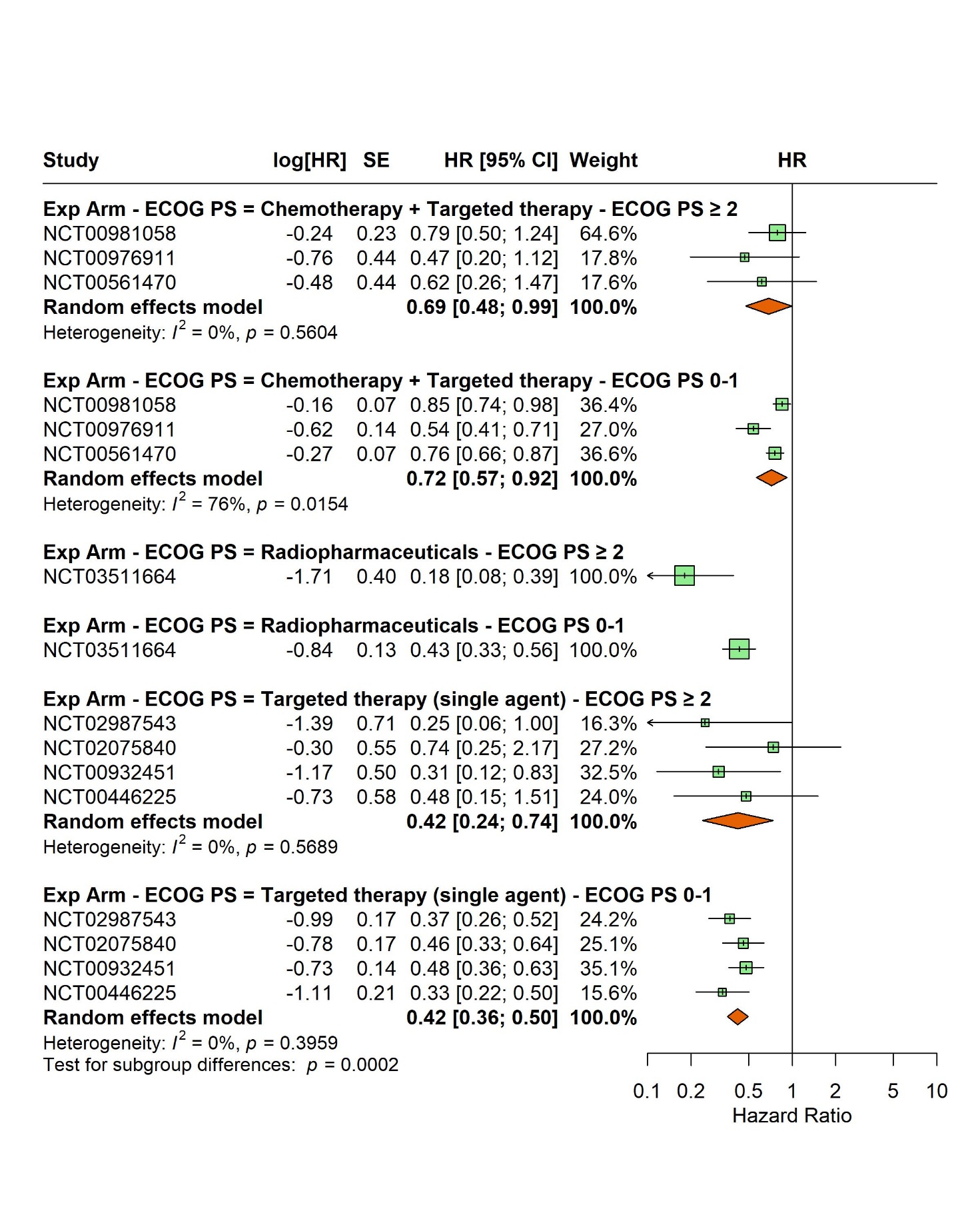


**Figure S16 -** Subgroup analysis of OS according to PS and drug class.


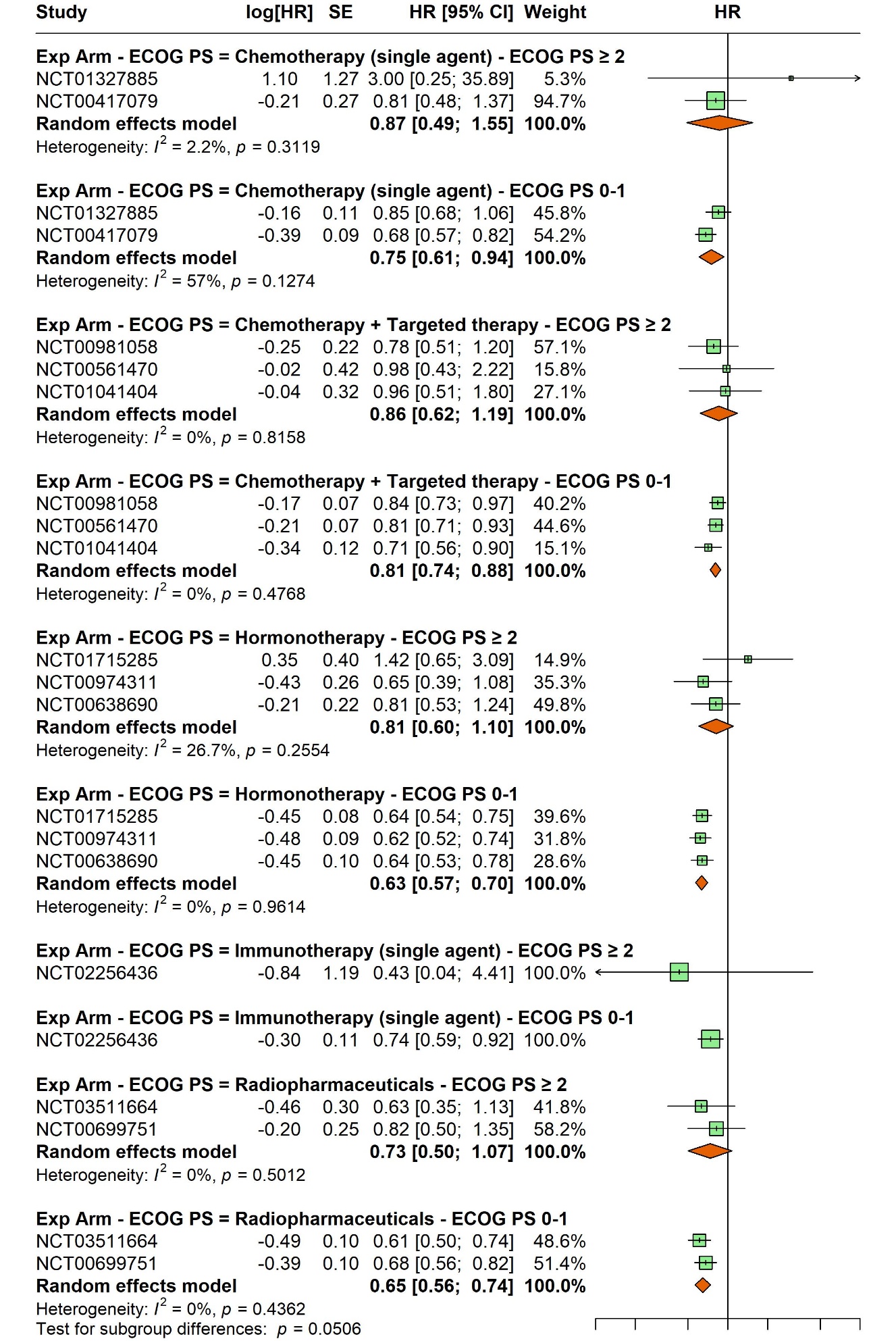


**Figure S17 -** Forest plot of pooled HRs for PFS.


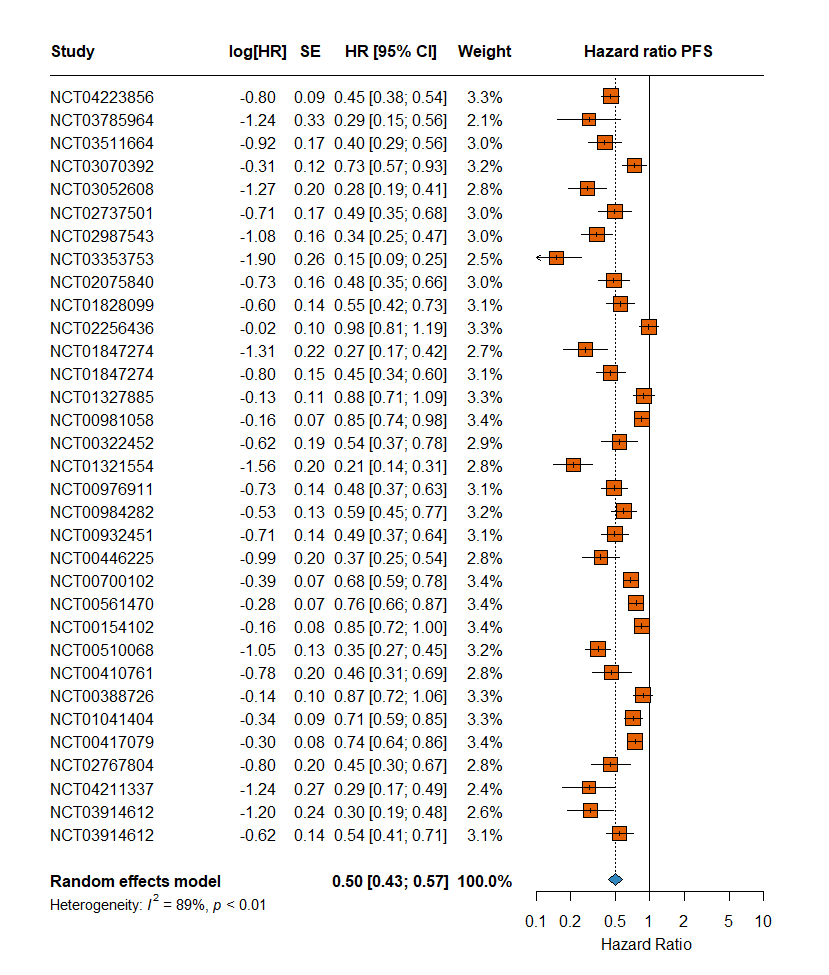


**Table S7** - Meta-regression model for PFS including the “total number of study participants”.

| **Model** | **Variable^a^** | **Estimate (β)** | **p-value** | **95% CI** | |
| --- | --- | --- | --- | --- | --- |
| Multivariable  *(n = 27)* | Intercept | -1.5273 | <.0001 | -1.9626 | -1.0921 |
|  | PS 1 participants | 0.0121 | 0.0544 | -0.0002 | 0.0244 |
|  | PS 2 participants | -0.0125 | 0.621 | -0.0622 | 0.0371 |
|  | **Total participants** | **0.0008** | **0.0002** | **0.0004** | **0.0012** |

*^a^Proportion of participants enrolled in clinical trials according to performance status.*

**Table S8** - Meta-regression models for PFS using arcsine-transformed proportions.

| **Model** | **Variable^a^** | **Estimate (β)** | **SE** | **z-value** | **p-value** | **95% CI** |
| --- | --- | --- | --- | --- | --- | --- |
| Univariable  *(n = 27)* | Intercept | -0.1771 | 0.5178 | -0.342 | 0.7323 | -1.1919 0.8377 |
|  | Arcsin(PS 0 participants) | -0.6497 | 0.6384 | -1.0178 | 0.3088 | -1.9009 0.6015 |
| Univariable  *(n = 27)* | Intercept | -1.6381 | 0.3233 | -5.0662 | <0.0001 | -2.2718 -1.0044 |
|  | **Arcsin(PS 1 participants)** | **1.3618** | **0.4506** | **3.0223** | **0.0025** | **0.4787 2.2450** |
| Univariable  *(n = 33)* | Intercept | -0.9839 | 0.1782 | -5.5216 | <0.0001 | -1.3332 -0.6347 |
|  | Arcsin(PS 2 participants) | 1.426 | 0.8166 | 1.7463 | 0.0808 | -0.1745 3.0264 |
| Multivariable  *(n = 27)* | Intercept | -1.675 | 0.336 | -4.985 | <0.0001 | -2.3336 -1.0164 |
|  | **Arcsin(PS 1 participants)** | **1.546** | **0.6052** | **2.554** | **0.0106** | **0.3598 2.7322** |
|  | Arcsin(PS 2 participants) | 0.501 | 1.0891 | 0.46 | 0.6455 | -2.6356 1.6335 |
| Multivariable  *(n = 27)* | Intercept | -1.7469 | 0.2805 | -6.2278 | <0.0001 | -2.2967 -1.1972 |
|  | **Arcsin(PS 1 participants)** | **1.1057** | **0.5115** | **2.1618** | **0.0306** | **0.1022 2.1081** |
|  | Arcsin(PS 2 participants) | -0.4749 | 0.8825 | -0.5381 | 0.5905 | -2.2045 1.2548 |
|  | **Total participants** | **0.0008** | **0.0002** | **3.8431** | **0.0001** | **0.0004 0.0011** |

*^a^Proportion of participants enrolled in clinical trials according to performance status.*

**Figure S18 -** Forest plot of pooled HRs for OS.


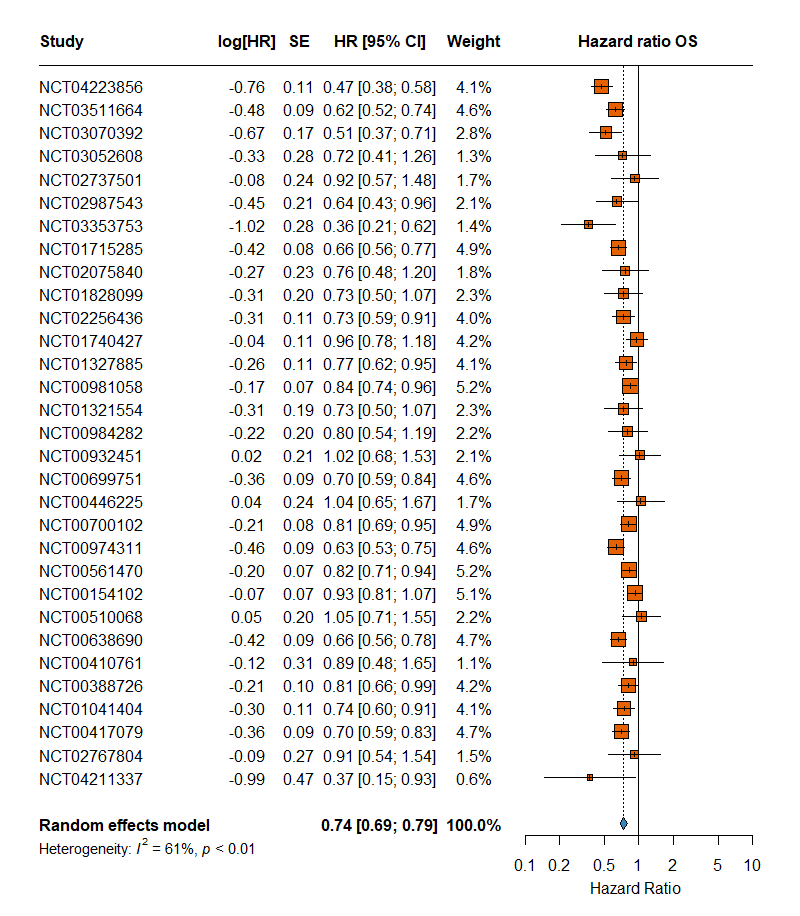


**Table S9** - Meta-regression model for OS including the “total number of study participants”.

| **Model** | **Variable^a^** | **Estimate (β)** | **p-value** | **95% CI** | |
| --- | --- | --- | --- | --- | --- |
| Multivariable  *(n = 23)* | Intercept | -0.6126 | 0.0054 | -1.0439 | -0.1813 |
|  | PS 1 participants | 0.0054 | 0.3247 | -0.0053 | 0.016 |
|  | PS 2 participants | 0.0094 | 0.6094 | -0.0267 | 0.0455 |
|  | Total participants | 0.0001 | 0.6817 | -0.0002 | 0.0004 |

*^a^Proportion of participants enrolled in clinical trials according to performance status.*

**Table S10** - Meta-regression models for OS using arcsine-transformed proportions.

| **Model** | **Variable^a^** | **Estimate (β)** | **SE** | **z-value** | **p-value** | **95% CI** | |
| --- | --- | --- | --- | --- | --- | --- | --- |
| Univariable  *(n = 23)* | Intercept | -0.147 | 0.3172 | -0.4635 | 0.643 | -0.7688 | 0.4747 |
|  | Arcsin(PS 0 participants) | -0.1667 | 0.419 | -0.3978 | 0.6908 | -0.9878 | 0.6545 |
| Univariable  *(n = 23)* | Intercept | -0.7729 | 0.2431 | -3.1791 | 0.0015 | -1.2495 | 0.2964 |
|  | **Arcsin(PS 1 participants)** | **0.6724** | **0.3188** | **2.1091** | **0.0349** | **0.0476** | **1.2972** |
| Univariable  *(n = 31)* | Intercept | -0.4776 | 0.4891 | -1.5993 | <.0001 | -0.7021 | -0.2531 |
|  | Arcsin(PS 2 participants) | -0.7823 | 0.4891 | -1.5993 | 0.1097 | -0.1764 | 1.7410 |
| Multivariable  *(n = 23)* | Intercept | -0.7307 | 0.2548 | -2.8674 | 0.0041 | -1.2301 | -0.2312 |
|  | Arcsin(PS 1 participants) | 0.4395 | 0.443 | 0.992 | 0.3212 | -0.4288 | 1.3077 |
|  | Arcsin(PS 2 participants) | 0.6301 | 0.7896 | 0.798 | 0.4249 | -0.9175 | 2.1778 |
| Multivariable  *(n = 23)* | Intercept | -0.7457 | 0.2612 | -2.8546 | 0.0043 | -1.2577 | -0.2337 |
|  | Arcsin(PS 1 participants) | 0.4101 | 0.4617 | 0.8883 | 0.3744 | -0.4948 | 1.5150 |
|  | Arcsin(PS 2 participants) | 0.6595 | 0.8097 | 0.8144 | 0.4154 | -0.9276 | 2.2466 |
|  | Total participants | 0 | 0.0002 | 0.2971 | 0.7664 | -0.0003 | 0.0004 |

*^a^Proportion of participants enrolled in clinical trials according to performance status.*

1. **SAFETY OUTCOMES**

**Figure S19** - Forest plot of pooled proportions for any-grade AEs.


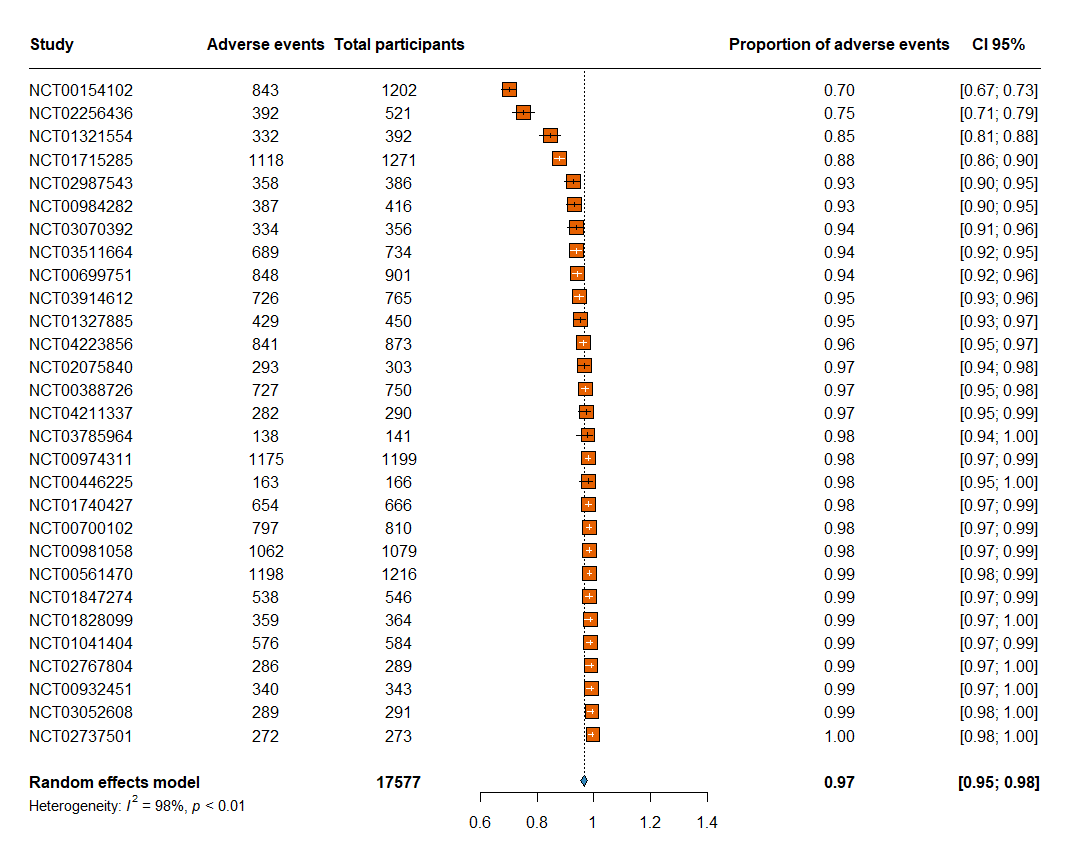


**Table S11** - Meta-regression models for any-grade AEs using arcsine-transformed proportions.

| **Model** | **Variable^a^** | **Estimate (β)** | **SE** | **t-value** | **p-value** | **95% CI** | |
| --- | --- | --- | --- | --- | --- | --- | --- |
| Univariable  *(n = 23)* | Intercept | 4.3813 | 1.3038 | 3.3604 | 0.003 | 1.6699 | 7.098 |
|  | Arcsin(PS 0 participants) | -1.2107 | 1.6596 | -0.7295 | 0.4737 | -4.6620 | 2.245 |
| Univariable  *(n = 23)* | Intercept | 2.3435 | 1.4961 | 1.5664 | 0.1322 | -0.7679 | 5.4549 |
|  | Arcsin(PS 1 participants) | 1.4793 | 1.9862 | 0.7448 | 0.4646 | -2.6511 | 5.6097 |
| Univariable  *(n = 29)* | Intercept | 2.8714 | 0.5153 | 5.5724 | <.0001 | 1.8141 | 3.9287 |
|  | Arcsin(PS 2 participants) | 2.4728 | 2.2876 | 1.0809 | 0.2893 | -2.2211 | 7.1667 |
| Multivariable  *(n = 23)* | Intercept | 2.7051 | 1.7276 | 1.5658 | 0.1331 | -0.8985 | 6.3088 |
|  | Arcsin(PS 1 participants) | 0.6036 | 2.8997 | 0.2082 | 0.8372 | -5.4452 | 6.6523 |
|  | Arcsin(PS 2 participants) | 1.4826 | 3.6003 | 0.4118 | 0.6849 | -6.0274 | 8.9926 |

*^a^Proportion of participants enrolled in clinical trials according to performance status.*

**Figure S20** - Forest plot of pooled proportions for high-grade AEs.


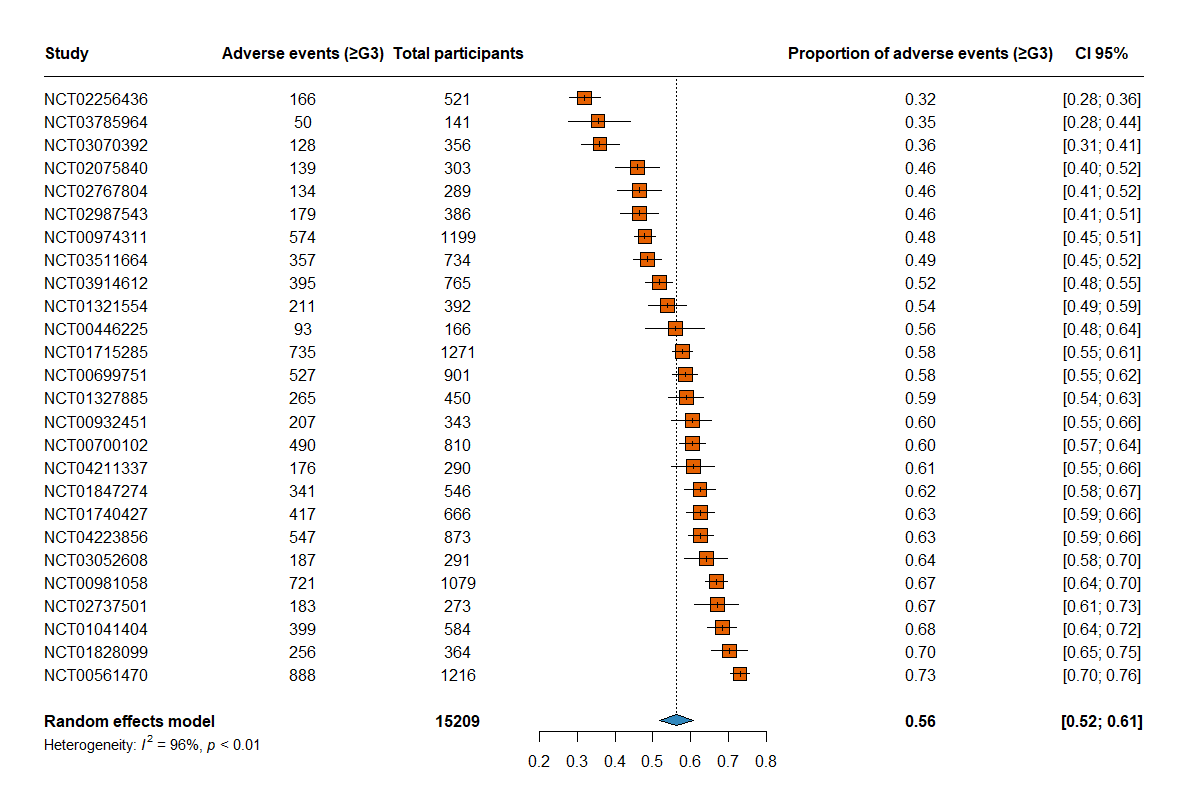


**Table S12** - Meta-regression models for high-grade AEs using arcsine-transformed proportions.

| **Model** | **Variable^a^** | **Estimate (β)** | **SE** | **t-value** | **p-value** | **95% CI** | |
| --- | --- | --- | --- | --- | --- | --- | --- |
| Univariable  *(n = 20)* | Intercept | 1.0393 | 0.5561 | 1.869 | 0.078 | -0.1290 | 2.2077 |
|  | Arcsin(PS 0 participants) | -0.9963 | 0.7153 | -1.3929 | 0.1806 | -2.4991 | 0.5064 |
| Univariable  *(n = 20)* | Intercept | -0.7398 | 0.6471 | -1.1433 | 0.2679 | -2.0993 | 0.6197 |
|  | Arcsin(PS 1 participants) | 1.3498 | 0.8478 | 1.5921 | 0.1288 | -0.4314 | 3.1310 |
| Univariable  *(n = 26)* | Intercept | -0.0083 | 0.2083 | -0.04 | 0.9684 | -0.4382 | 0.4215 |
|  | Arcsin(PS 2 participants) | 1.2488 | 0.9183 | 1.3599 | 0.1865 | -0.6465 | 3.1441 |
| Multivariable  *(n = 20)* | Intercept | -0.5844 | 0.7751 | -0.7539 | 0.4612 | -2.2198 | 1.0510 |
|  | Arcsin(PS 1 participants) | 0.9977 | 1.2904 | 0.7732 | 0.45 | -1.7248 | 3.7202 |
|  | Arcsin(PS 2 participants) | 0.5675 | 1.5735 | 0.3607 | 0.7228 | -2.7523 | 3.8874 |

*^a^Proportion of participants enrolled in clinical trials according to performance status.*

**Figure S21** - Forest plot of pooled proportions for SAEs.


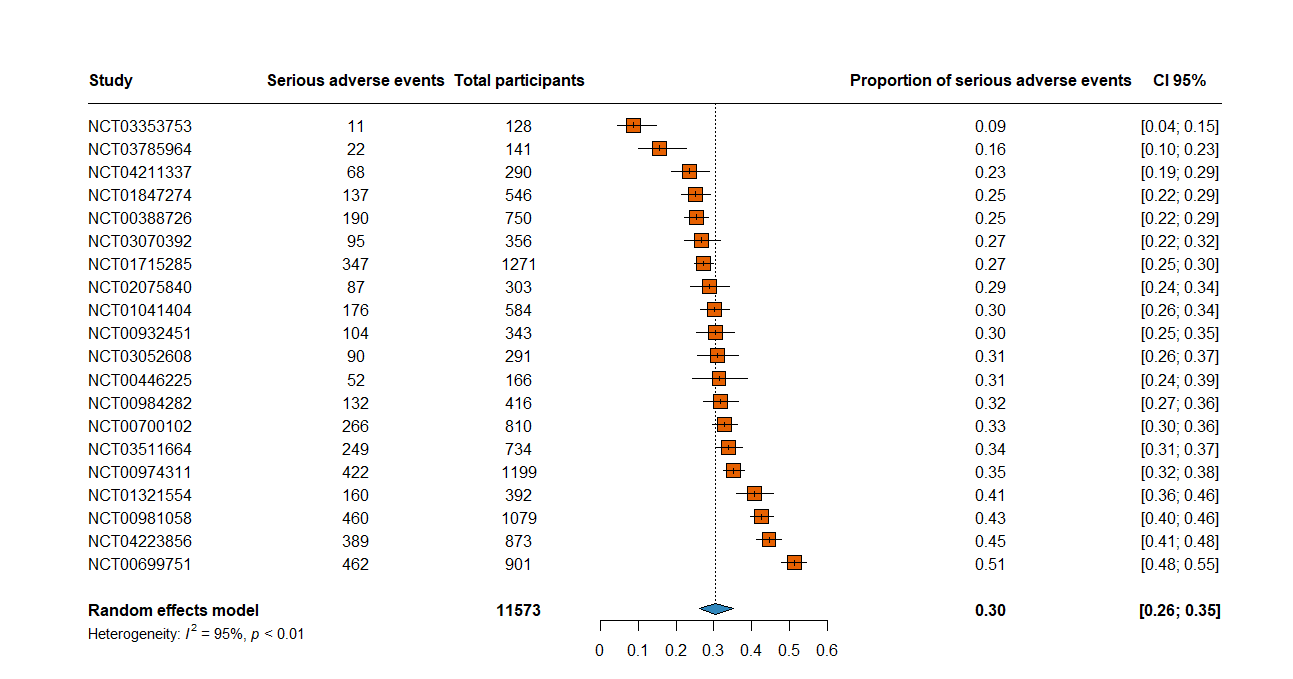


**Table S13** - Meta-regression models for SAEs using arcsine-transformed proportions.

| **Model** | **Variable^a^** | **Estimate (β)** | **SE** | **t-value** | **p-value** | **95% CI (Lower – Upper)** | |
| --- | --- | --- | --- | --- | --- | --- | --- |
| Univariable  *(n = 15)* | Intercept | 0.2737 | 0.5866 | 0.4667 | 0.6484 | -0.9934 | 1.5409 |
|  | Arcsin(PS 0 participants) | -1.5099 | 0.7546 | -2.001 | 0.0667 | -3.1400 | 0.1203 |
| Univariable  *(n = 15)* | Intercept | -2.338 | 0.3293 | -7.0989 | <.0001 | -3.0495 | -1.6265 |
|  | **Arcsin(PS 1 participants)** | **2.0874** | **0.444** | **4.701** | **0.0004** | **1.1281** | **3.0467** |
| Univariable  *(n = 20)* | Intercept | -1.3748 | 0.1791 | -7.6762 | <.0001 | -1.7511 | -0.9985 |
|  | **Arcsin(PS 2 participants)** | **2.6049** | **0.7428** | **3.5068** | **0.0025** | **1.0443** | **4.1655** |
| Multivariable  *(n = 15)* | Intercept | -2.3093 | 0.3673 | -6.2872 | <.0001 | -3.1096 | -1.5090 |
|  | **Arcsin(PS 1 participants)** | **1.991** | **0.7096** | **2.8057** | **0.0159** | **0.4448** | **3.5371** |
|  | Arcsin(PS 2 participants) | 0.2008 | 1.1555 | 0.1738 | 0.865 | -2.3169 | 2.7185 |

*^a^Proportion of participants enrolled in clinical trials according to performance status.*

**Figure S22** - Forest plot of pooled proportions for dose interruptions.


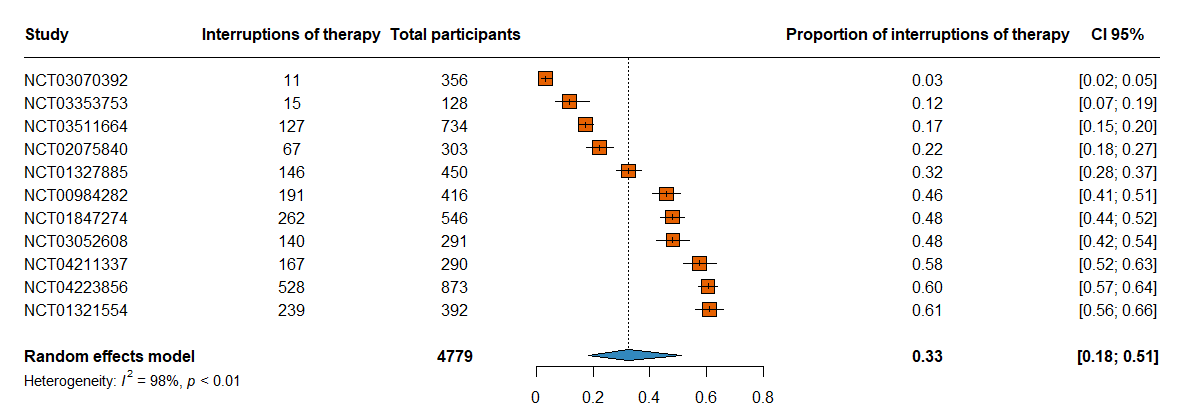


**Table S14** - Meta-regression models for dose interruptions using arcsine-transformed proportions.

| **Model** | **Variable^a^** | **Estimate (β)** | **SE** | **t-value** | **p-value** | **95% CI** | |
| --- | --- | --- | --- | --- | --- | --- | --- |
| Univariable  *(n = 9)* | Intercept | 0.149 | 2.4027 | 0.062 | 0.9523 | -5.5323 | 5.8304 |
|  | Arcsin(PS 0 participants) | -1.1281 | 2.9184 | -0.3866 | 0.7106 | -8.0291 | 5.7728 |
| Univariable  *(n = 9)* | Intercept | -2.2566 | 0.9253 | -2.4387 | 0.0448 | -4.4447 | -0.0685 |
|  | Arcsin(PS 1 participants) | 2.3634 | 1.3592 | 1.7389 | 0.1256 | -0.8505 | 5.5773 |
| Univariable  *(n = 11)* | Intercept | -1.1504 | 0.6472 | -1.7774 | 0.1092 | -2.6146 | 0.3137 |
|  | Arcsin(PS 2 participants) | 2.8305 | 3.6928 | 0.7665 | 0.463 | -5.5233 | 11.1842 |
| Multivariable  *(n = 9)* | Intercept | -2.3839 | 0.9647 | -2.4712 | 0.0484 | -4.7443 | -0.0235 |
|  | Arcsin(PS 1 participants) | 3.1053 | 2.2249 | 1.3957 | 0.2123 | -2.3389 | 8.5495 |
|  | Arcsin(PS 2 participants) | -2.6405 | 6.3067 | -0.4187 | 0.69 | -18.0723 | 12.7913 |

*^a^Proportion of participants enrolled in clinical trials according to performance status.*

**Figure S23** - Forest plot of pooled proportions for dose reductions.


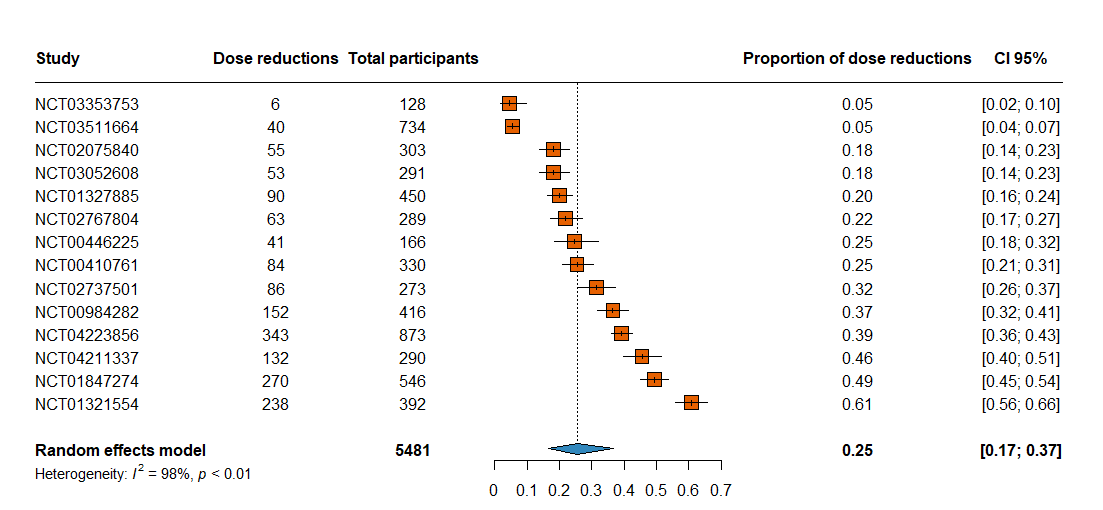


**Table S15** - Meta-regression models for dose reductions using arcsine-transformed proportions.

| **Model** | **Variable^a^** | **Estimate (β)** | **SE** | **t-value** | **p-value** | **95% CI** | |
| --- | --- | --- | --- | --- | --- | --- | --- |
| Univariable  *(n =11)* | Intercept | -3.6795 | 1.0503 | -3.5033 | 0.0067 | -6.0554 | -1.3036 |
|  | **Arcsin(PS 0 participants)** | **3.4536** | **1.3344** | **2.5882** | **0.0293** | **0.4351** | **6.4721** |
| Univariable  *(n = 11)* | Intercept | -2.0786 | 0.677 | -3.0705 | 0.0133 | -3.6100 | -0.5472 |
|  | Arcsin(PS 1 participants) | 1.5512 | 0.9357 | 1.6577 | 0.1318 | -0.5656 | 3.6680 |
| Univariable  *(n = 14)* | Intercept | -0.8324 | 0.5334 | -1.5606 | 0.1446 | -1.9946 | 0.3298 |
|  | Arcsin(PS 2 participants) | -1.2745 | 2.5252 | -0.5047 | 0.6229 | -6.7764 | 4.2273 |
| Multivariable  *(n = 11)* | Intercept | -2.2397 | 0.5958 | -3.7592 | 0.0056 | -3.6136 | -0.8658 |
|  | **Arcsin(PS 1 participants)** | **3.0435** | **1.0966** | **2.7754** | **0.0241** | **0.5147** | **5.5722** |
|  | Arcsin(PS 2 participants) | -4.8637 | 2.2697 | -2.1428 | 0.0645 | -10.0976 | 0.3703 |

*^a^Proportion of participants enrolled in clinical trials according to performance status.*

**Figure S24** - Forest plot of pooled proportions for dose discontinuations.


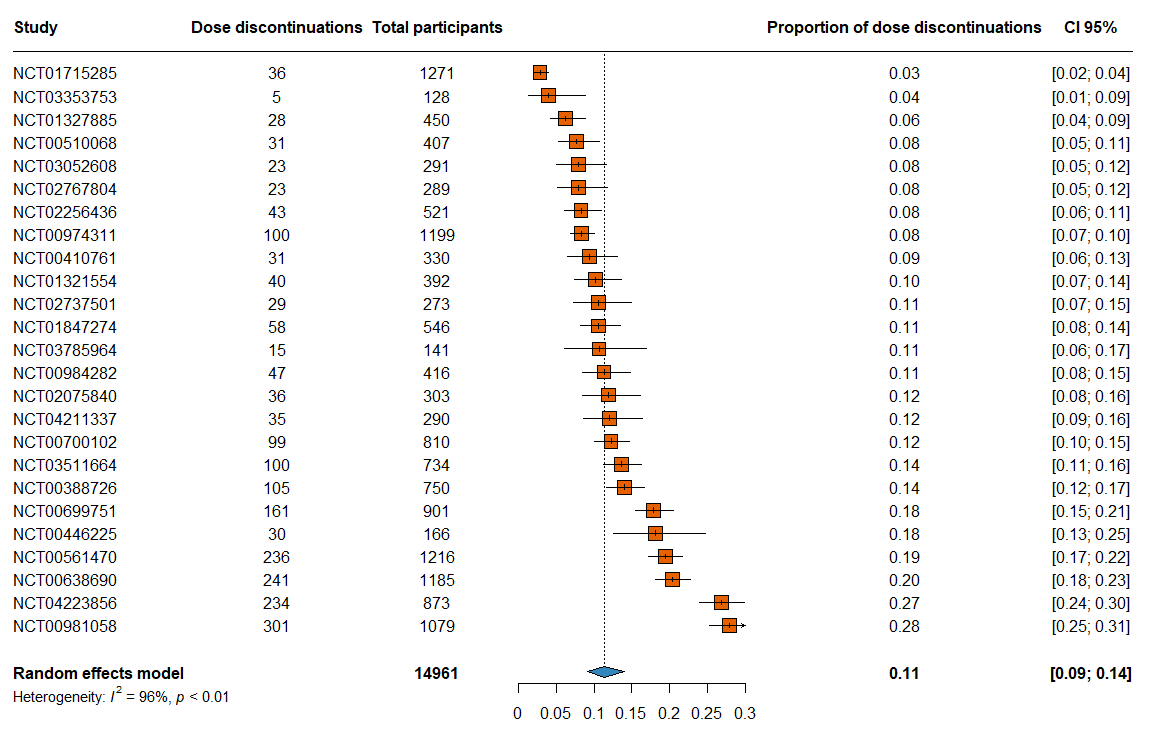


**Table S16** - Meta-regression models for dose discontinuations using arcsine-transformed proportions.

| **Model** | **Variable^a^** | **Estimate (β)** | **SE** | **t-value** | **p-value** | **95% CI** | |
| --- | --- | --- | --- | --- | --- | --- | --- |
| Univariable  *(n = 19)* | Intercept | -1.17 | 0.6521 | -1.7943 | 0.0906 | -2.5457 | 0.2058 |
|  | Arcsin(PS 0 participants) | -1.0542 | 0.8374 | -1.2588 | 0.2251 | -2.8210 | 0.7127 |
| Univariable  *(n =19)* | Intercept | -3.0898 | 0.4806 | -6.4291 | <.0001 | -4.1037 | -2.0758 |
|  | **Arcsin(PS 1 participants)** | **1.5419** | **0.6433** | **2.3969** | **0.0283** | **0.1847** | **2.8991** |
| Univariable  *(n = 25)* | Intercept | -2.6116 | 0.2616 | -9.9828 | <.0001 | -3.1528 | -2.0704 |
|  | **Arcsin(PS 2 participants)** | **2.6633** | **1.1408** | **2.3346** | **0.0287** | **0.3034** | **5.0232** |
| Multivariable  *(n = 19)* | Intercept | -2.9534 | 0.4825 | -6.1213 | <.0001 | -3.9762 | -1.9306 |
|  | Arcsin(PS 1 participants) | 0.941 | 0.8349 | 1.127 | 0.2764 | -0.8290 | 2.7110 |
|  | Arcsin(PS 2 participants) | 1.5446 | 1.4325 | 1.0783 | 0.2969 | -1.4922 | 4.5815 |

*^a^Proportion of participants enrolled in clinical trials according to performance status.*

**Figure S25** - Forest plot of pooled proportions for AE-related deaths.


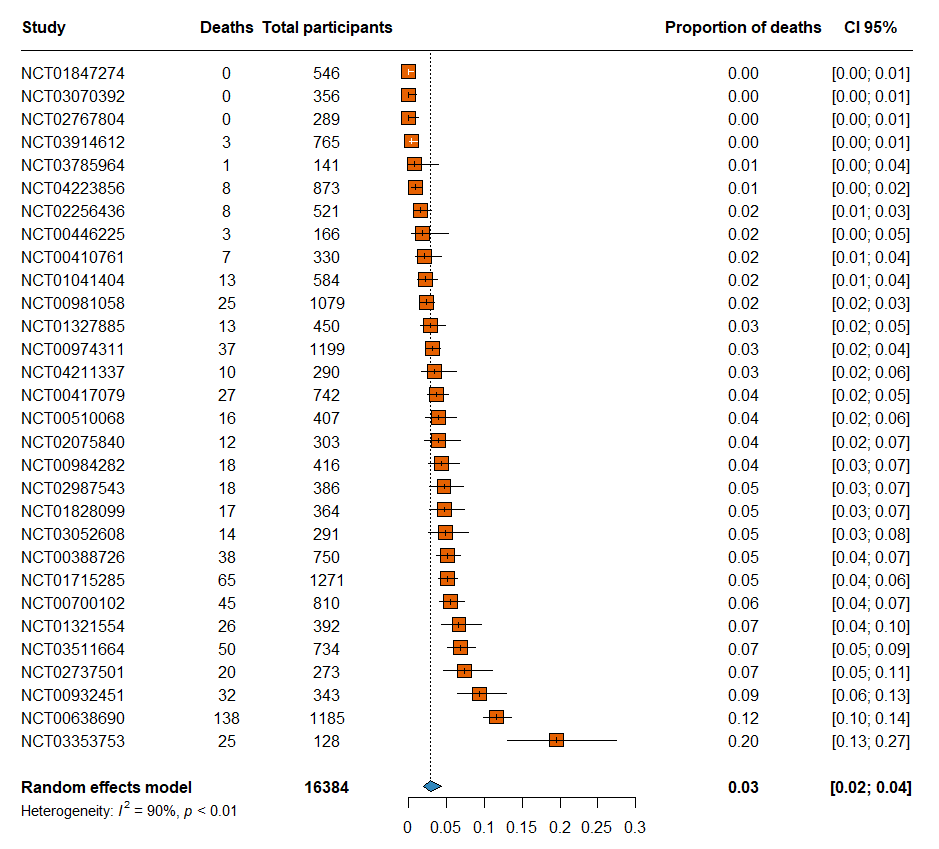


**Table S17** - Meta-regression models for AE-related deaths using arcsine-transformed proportions.

| **Model** | **Variable^a^** | **Estimate (β)** | **SE** | **t-value** | **p-value** | **95% CI** | |
| --- | --- | --- | --- | --- | --- | --- | --- |
| Univariable  *(n = 22)* | Intercept | 0.3827 | 1.2704 | 0.3012 | 0.7663 | -2.2673 | 3.0326 |
|  | **Arcsin(PS 0 participants)** | **-5.1412** | **1.6395** | **-3.1358** | **0.0052** | **-8.5611** | **-1.7213** |
| Univariable  *(n = 22)* | Intercept | -3.5467 | 0.9319 | -3.8059 | 0.0011 | -5.4907 | -1.6028 |
|  | Arcsin(PS 1 participants) | -0.111 | 1.2678 | -0.0875 | 0.9311 | -2.7556 | 2.5336 |
| Univariable  *(n = 30)* | Intercept | -4.3837 | 0.5238 | -8.3698 | <.0001 | -5.4566 | -3.3109 |
|  | Arcsin(PS 2 participants) | 4.1112 | 2.2438 | 1.8323 | 0.0776 | -0.4849 | 8.7073 |
| Multivariable  *(n = 22)* | Intercept | -3.0764 | 0.8264 | -3.7228 | 0.0014 | -4.8061 | -1.3468 |
|  | Arcsin(PS 1 participants) | -3.1135 | 1.5755 | -1.9762 | 0.0628 | -6.4110 | 0.1841 |
|  | **Arcsin(PS 2 participants)** | **8.7462** | **3.4579** | **2.5293** | **0.0204** | **1.5087** | **15.9837** |

*^a^Proportion of participants enrolled in clinical trials according to performance status.*

1. **ANALYSES OF MULTICOLLINEARITY**

**Table S16** - Variance inflation factors for covariates included in meta-regression models.

| **Outcome** | **Model** | **Variable*** | **VIF** |
| --- | --- | --- | --- |
| **PFS** | Multivariable | PS 1 participants | 1.64 |
|  |  | PS 2 participants | 1.64 |
|  | Multivariable | PS 1 participants | 1.81 |
|  |  | PS 2 participants | 1.67 |
|  |  | Total participants | 1.12 |
|  | *Multivariable (Arcsin transformation)* | *PS 1 participants* | *1.81* |
|  |  | *PS 2 participants* | *1.81* |
|  | *Multivariable (Arcsin transformation)* | *PS 1 participants* | *1.92* |
|  |  | *PS 2 participants* | *1.81* |
|  |  | *Total participants* | *1.10* |
| **OS** | Multivariable | PS 1 participants | 1.59 |
|  |  | PS 2 participants | 1.59 |
|  | Multivariable | PS 1 participants | 1.72 |
|  |  | PS 2 participants | 1.62 |
|  |  | Total participants | 1.09 |
|  | *Multivariable (Arcsin transformation)* | *PS 1 participants* | *2.05* |
|  |  | *PS 2 participants* | *2.05* |
|  | *Multivariable (Arcsin transformation)* | *PS 1 participants* | *2.21* |
|  |  | *PS 2 participants* | *2.07* |
|  |  | *Total participants* | *1.10* |
| **Any-grade AEs** | Multivariable | PS 1 participants | 1.80 |
|  |  | PS 2 participants | 1.80 |
|  | *Multivariable (Arcsin transformation)* | *PS 1 participants* | *2.18* |
|  |  | *PS 2 participants* | *2.18* |
| **High-grade AEs** | Multivariable | PS 1 participants | 1.82 |
|  |  | PS 2 participants | 1.82 |
|  | *Multivariable (Arcsin transformation)* | *PS 1 participants* | *2.33* |
|  |  | *PS 2 participants* | *2.33* |
| **SAEs** | Multivariable | PS 1 participants | 2.27 |
|  |  | PS 2 participants | 2.27 |
|  | *Multivariable (Arcsin transformation)* | *PS 1 participants* | *2.58* |
|  |  | *PS 2 participants* | *2.58* |
| **Dose interruptions** | Multivariable | PS 1 participants | 3.37 |
|  |  | PS 2 participants | 3.37 |
|  | *Multivariable (Arcsin transformation)* | *PS 1 participants* | *2.78* |
|  |  | *PS 2 participants* | *2.78* |
| **Dose reductions** | Multivariable | PS 1 participants | 1.52 |
|  |  | PS 2 participants | 1.52 |
|  | *Multivariable (Arcsin transformation)* | *PS 1 participants* | *1.99* |
|  |  | *PS 2 participants* | *1.99* |
| **Dose discontinuations** | Multivariable | PS 1 participants | 1.61 |
|  |  | PS 2 participants | 1.61 |
|  | *Multivariable (Arcsin transformation)* | *PS 1 participants* | *1.90* |
|  |  | *PS 2 participants* | *1.90* |
| **AE-related deaths** | Multivariable | PS 1 participants | 1.67 |
|  |  | PS 2 participants | 1.67 |
|  | *Multivariable (Arcsin transformation)* | *PS 1 participants* | *2.00* |
|  |  | *PS 2 participants* | *2.00* |

*^a^Proportion of participants enrolled in clinical trials according to performance status.*

1. **R PACKAGES USED**

- Utilities and data wrangling: readxl, writexl, dplyr.
- Data visualization: ggplot2, car.
- Descriptive statistics: summarytools.
- Meta-analysis and meta-regression: meta, metaphor, car.

1. **LIST OF ABBREVIATIONS**

- 95% Confidence Interval (95%CI)
- Adverse Event (AE)
- Androgen Deprivation Therapy (ADT)
- Bladder Cancer (BLC)
- Breast Cancer (BrC)
- Colorectal Cancer (CRC)
- Endometrial Cancer (EC)
- Eastern Cooperative Oncology Group (ECOG)
- Gastric Cancer/Gastroesophageal Cancer (GC)
- Gastrointestinal (GI)
- Gastrointestinal Stromal Tumors (GIST)
- Genitourinary (GU)
- Hazard Ratio (HR)
- Imaging-based Progression-Free Survival (ibPFS)
- Intravenous (IV)
- Neuroendocrine Tumors (NT)
- Non-Small Cell Lung Cancer (NSCLC)
- Per Os (PO)
- Perfomance Status (PS)
- Progression-free Survival (PFS)
- Prostate Cancer (PCa)
- Ovarian Cancer (OC)
- Overall Survival (OS)
- Standard Error (SE)
- Soft Tissue Sarcoma (STS)
- Thyroid Cancer (TC)
- Variance Inflaction Factor (VIF)
- World Health Organization (WHO)

1. **REFERENCES**

1. Horn L, Wang Z, Wu G, et al. Ensartinib vs Crizotinib for Patients With Anaplastic Lymphoma Kinase-Positive Non-Small Cell Lung Cancer: A Randomized Clinical Trial. *JAMA Oncol*. 2021;7(11):1617-1625. doi:10.1001/jamaoncol.2021.3523

2. Hadoux J, Elisei R, Brose MS, et al. Phase 3 Trial of Selpercatinib in Advanced RET-Mutant Medullary Thyroid Cancer. *N Engl J Med*. 2023;389(20):1851-1861. doi:10.1056/NEJMoa2309719

3. Eskander RN, Sill MW, Beffa L, et al. Pembrolizumab plus Chemotherapy in Advanced Endometrial Cancer. *N Engl J Med*. 2023;388(23):2159-2170. doi:10.1056/NEJMoa2302312

4. Powles T, Valderrama BP, Gupta S, et al. Enfortumab Vedotin and Pembrolizumab in Untreated Advanced Urothelial Cancer. *N Engl J Med*. 2024;390(10):875-888. doi:10.1056/NEJMoa2312117

5. Gounder M, Ratan R, Alcindor T, et al. Nirogacestat, a γ-Secretase Inhibitor for Desmoid Tumors. *N Engl J Med*. 2023;388(10):898-912. doi:10.1056/NEJMoa2210140

6. Sartor O, de Bono J, Chi KN, et al. Lutetium-177-PSMA-617 for Metastatic Castration-Resistant Prostate Cancer. *N Engl J Med*. 2021;385(12):1091-1103. doi:10.1056/NEJMoa2107322

7. Nathan P, Hassel JC, Rutkowski P, et al. Overall Survival Benefit with Tebentafusp in Metastatic Uveal Melanoma. *N Engl J Med*. 2021;385(13):1196-1206. doi:10.1056/NEJMoa2103485

8. Shaw AT, Bauer TM, de Marinis F, et al. First-Line Lorlatinib or Crizotinib in Advanced ALK-Positive Lung Cancer. *N Engl J Med*. 2020;383(21):2018-2029. doi:10.1056/NEJMoa2027187

9. Camidge DR, Kim HR, Ahn MJ, et al. Brigatinib Versus Crizotinib in Advanced ALK Inhibitor-Naive ALK-Positive Non-Small Cell Lung Cancer: Second Interim Analysis of the Phase III ALTA-1L Trial. *J Clin Oncol*. 2020;38(31):3592-3603. doi:10.1200/JCO.20.00505

10. de Bono J, Mateo J, Fizazi K, et al. Olaparib for Metastatic Castration-Resistant Prostate Cancer. *N Engl J Med*. 2020;382(22):2091-2102. doi:10.1056/NEJMoa1911440

11. Blay JY, Serrano C, Heinrich MC, et al. Ripretinib in patients with advanced gastrointestinal stromal tumours (INVICTUS): a double-blind, randomised, placebo-controlled, phase 3 trial. *Lancet Oncol*. 2020;21(7):923-934. doi:10.1016/S1470-2045(20)30168-6

12. Fizazi K, Tran N, Fein L, et al. Abiraterone acetate plus prednisone in patients with newly diagnosed high-risk metastatic castration-sensitive prostate cancer (LATITUDE): final overall survival analysis of a randomised, double-blind, phase 3 trial. *Lancet Oncol*. 2019;20(5):686-700. doi:10.1016/S1470-2045(19)30082-8

13. Peters S, Camidge DR, Shaw AT, et al. Alectinib versus Crizotinib in Untreated ALK-Positive Non-Small-Cell Lung Cancer. *N Engl J Med*. 2017;377(9):829-838. doi:10.1056/NEJMoa1704795

14. Soria JC, Tan DSW, Chiari R, et al. First-line ceritinib versus platinum-based chemotherapy in advanced ALK-rearranged non-small-cell lung cancer (ASCEND-4): a randomised, open-label, phase 3 study. *Lancet*. 2017;389(10072):917-929. doi:10.1016/S0140-6736(17)30123-X

15. Bellmunt J, de Wit R, Vaughn DJ, et al. Pembrolizumab as Second-Line Therapy for Advanced Urothelial Carcinoma. *N Engl J Med*. 2017;376(11):1015-1026. doi:10.1056/NEJMoa1613683

16. Slamon DJ, Diéras V, Rugo HS, et al. Overall Survival With Palbociclib Plus Letrozole in Advanced Breast Cancer. *J Clin Oncol*. 2024;42(9):994-1000. doi:10.1200/JCO.23.00137

17. Mirza MR, Monk BJ, Herrstedt J, et al. Niraparib Maintenance Therapy in Platinum-Sensitive, Recurrent Ovarian Cancer. *N Engl J Med*. 2016;375(22):2154-2164. doi:10.1056/NEJMoa1611310

18. Schöffski P, Chawla S, Maki RG, et al. Eribulin versus dacarbazine in previously treated patients with advanced liposarcoma or leiomyosarcoma: a randomised, open-label, multicentre, phase 3 trial. *Lancet*. 2016;387(10028):1629-1637. doi:10.1016/S0140-6736(15)01283-0

19. Thatcher N, Hirsch FR, Luft AV, et al. Necitumumab plus gemcitabine and cisplatin versus gemcitabine and cisplatin alone as first-line therapy in patients with stage IV squamous non-small-cell lung cancer (SQUIRE): an open-label, randomised, controlled phase 3 trial. *Lancet Oncol*. 2015;16(7):763-774. doi:10.1016/S1470-2045(15)00021-2

20. Wu YL, Saijo N, Thongprasert S, et al. Efficacy according to blind independent central review: Post-hoc analyses from the phase III, randomized, multicenter, IPASS study of first-line gefitinib versus carboplatin/paclitaxel in Asian patients with EGFR mutation-positive advanced NSCLC. *Lung Cancer*. 2017;104:119-125. doi:10.1016/j.lungcan.2016.11.022

21. Schlumberger M, Tahara M, Wirth LJ, et al. Lenvatinib versus placebo in radioiodine-refractory thyroid cancer. *N Engl J Med*. 2015;372(7):621-630. doi:10.1056/NEJMoa1406470

22. Husain A, Wang Y, Hanker LC, et al. Independent radiologic review of AURELIA, a phase 3 trial of bevacizumab plus chemotherapy for platinum-resistant recurrent ovarian cancer. *Gynecol Oncol*. 2016;142(3):465-470. doi:10.1016/j.ygyno.2016.05.011

23. Brose MS, Nutting CM, Jarzab B, et al. Sorafenib in radioactive iodine-refractory, locally advanced or metastatic differentiated thyroid cancer: a randomised, double-blind, phase 3 trial. *Lancet*. 2014;384(9940):319-328. doi:10.1016/S0140-6736(14)60421-9

24. Shaw AT, Kim DW, Nakagawa K, et al. Crizotinib versus chemotherapy in advanced ALK-positive lung cancer. *N Engl J Med*. 2013;368(25):2385-2394. doi:10.1056/NEJMoa1214886

25. Parker C, Nilsson S, Heinrich D, et al. Alpha emitter radium-223 and survival in metastatic prostate cancer. *N Engl J Med*. 2013;369(3):213-223. doi:10.1056/NEJMoa1213755

26. Rosell R, Carcereny E, Gervais R, et al. Erlotinib versus standard chemotherapy as first-line treatment for European patients with advanced EGFR mutation-positive non-small-cell lung cancer (EURTAC): a multicentre, open-label, randomised phase 3 trial. *Lancet Oncol*. 2012;13(3):239-246. doi:10.1016/S1470-2045(11)70393-X

27. Bennouna J, Sastre J, Arnold D, et al. Continuation of bevacizumab after first progression in metastatic colorectal cancer (ML18147): a randomised phase 3 trial. *Lancet Oncol*. 2013;14(1):29-37. doi:10.1016/S1470-2045(12)70477-1

28. Scher HI, Fizazi K, Saad F, et al. Increased survival with enzalutamide in prostate cancer after chemotherapy. *N Engl J Med*. 2012;367(13):1187-1197. doi:10.1056/NEJMoa1207506

29. Van Cutsem E, Tabernero J, Lakomy R, et al. Addition of aflibercept to fluorouracil, leucovorin, and irinotecan improves survival in a phase III randomized trial in patients with metastatic colorectal cancer previously treated with an oxaliplatin-based regimen. *J Clin Oncol*. 2012;30(28):3499-3506. doi:10.1200/JCO.2012.42.8201

30. Van Cutsem E, Köhne CH, Hitre E, et al. Cetuximab and chemotherapy as initial treatment for metastatic colorectal cancer. *N Engl J Med*. 2009;360(14):1408-1417. doi:10.1056/NEJMoa0805019

31. Yao JC, Shah MH, Ito T, et al. Everolimus for advanced pancreatic neuroendocrine tumors. *N Engl J Med*. 2011;364(6):514-523. doi:10.1056/NEJMoa1009290

32. de Bono JS, Logothetis CJ, Molina A, et al. Abiraterone and increased survival in metastatic prostate cancer. *N Engl J Med*. 2011;364(21):1995-2005. doi:10.1056/NEJMoa1014618

33. Wells SA, Robinson BG, Gagel RF, et al. Vandetanib in patients with locally advanced or metastatic medullary thyroid cancer: a randomized, double-blind phase III trial. *J Clin Oncol*. 2012;30(2):134-141. doi:10.1200/JCO.2011.35.5040

34. Cortes J, O’Shaughnessy J, Loesch D, et al. Eribulin monotherapy versus treatment of physician’s choice in patients with metastatic breast cancer (EMBRACE): a phase 3 open-label randomised study. *Lancet*. 2011;377(9769):914-923. doi:10.1016/S0140-6736(11)60070-6

35. Bang YJ, Van Cutsem E, Feyereislova A, et al. Trastuzumab in combination with chemotherapy versus chemotherapy alone for treatment of HER2-positive advanced gastric or gastro-oesophageal junction cancer (ToGA): a phase 3, open-label, randomised controlled trial. *Lancet*. 2010;376(9742):687-697. doi:10.1016/S0140-6736(10)61121-X

36. de Bono JS, Oudard S, Ozguroglu M, et al. Prednisone plus cabazitaxel or mitoxantrone for metastatic castration-resistant prostate cancer progressing after docetaxel treatment: a randomised open-label trial. *Lancet*. 2010;376(9747):1147-1154. doi:10.1016/S0140-6736(10)61389-X
